# Direct evidence of the molecular basis for biological silicon transport

**DOI:** 10.1038/ncomms11926

**Published:** 2016-06-16

**Authors:** Michael J. Knight, Laura Senior, Bethany Nancolas, Sarah Ratcliffe, Paul Curnow

**Affiliations:** 1 School of Biochemistry, University of Bristol, Bristol BS8 1TD, UK; 2 BrisSynBio, Life Sciences Building, Tyndall Avenue, Bristol BS8 1TQ, UK

## Abstract

Diatoms are an important group of eukaryotic algae with a curious evolutionary innovation: they sheath themselves in a cell wall made largely of silica. The cellular machinery responsible for silicification includes a family of membrane permeases that recognize and actively transport the soluble precursor of biosilica, silicic acid. However, the molecular basis of silicic acid transport remains obscure. Here, we identify experimentally tractable diatom silicic acid transporter (SIT) homologues and study their structure and function *in vitro*, enabled by the development of a new fluorescence method for studying substrate transport kinetics. We show that recombinant SITs are Na^+^/silicic acid symporters with a 1:1 protein: substrate stoichiometry and *K*_M_ for silicic acid of 20 μM. Protein mutagenesis supports the long-standing hypothesis that four conserved GXQ amino acid motifs are important in SIT function. This marks a step towards a detailed understanding of silicon transport with implications for biogeochemistry and bioinspired materials.

## Abstract (web summary)

Diatoms sheath themselves in a self-made casing of silica, which requires the function of silicic acid transporters. Here, the authors identify versions of these transporters that are experimentally tractable, and develop a fluorescence method to study silicic acid transport *in vitro*.

## Graphical abstract

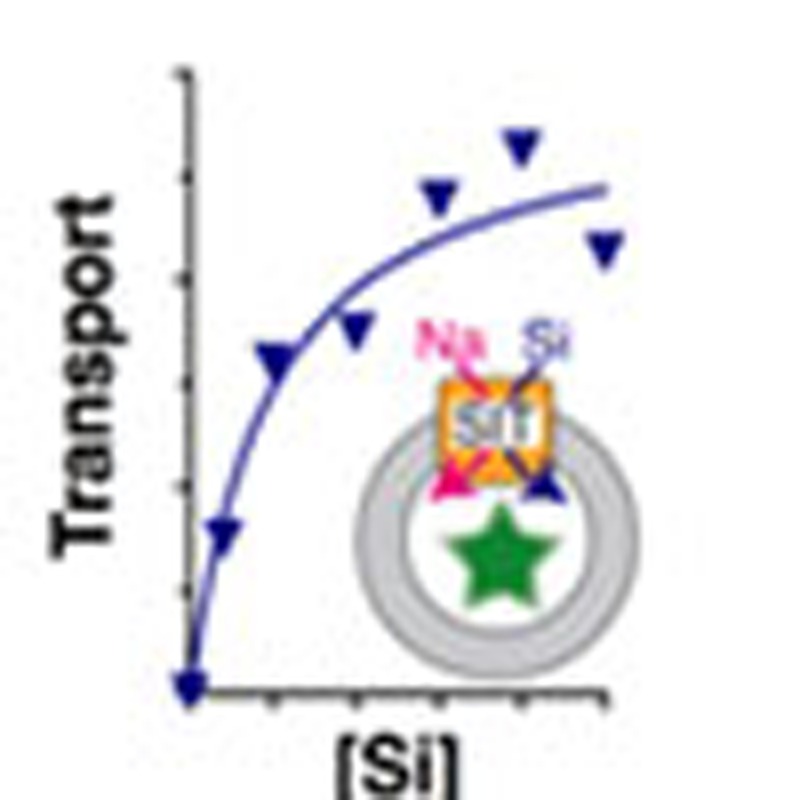

Diatoms (Bacillariophyceae) are unicellar eukaryotic algae. They are among the most important and diverse photosynthetic producers in the biosphere, with over 100,000 species distributed in oceans, lakes, rivers, wetlands and soils^1^. Diatoms are estimated to contribute nearly half of the total ocean primary production and as much as 20% of global photosynthetic carbon fixation^2^,^3^.

One unusual characteristic of diatoms is the presence of a silicified outer cell wall known as the frustule. This structure is essential to the survival of many (although not all) of the common diatoms and is formed from the polycondensation of a soluble precursor, silicic acid. Frustules are intricate and ornate hierarchical structures that are synthesized with control over the silica architecture from the nanoscale to the microscale, and the gross morphology of the frustule can vary markedly across different diatom species. Silicification occurs just after vegetative cell division within a dedicated intracellular compartment known as the silica deposition vesicle (SDV). This involves a collection of specialized genes whose expression is regulated either by cell cycle progression, environmental concentrations of silicic acid, or both^4^,^5^,^6^,^7^. The prevalent model^8^,^9^ is that, within the SDV, pre-organized biomacromolecules template and regulate the polymerization of silicic acid to silica^10^,^11^,^12^,^13^,^14^,^15^,^16^,^17^,^18^,^19^. This direct biological control over silica formation is augmented by cytoskeletal proteins that determine the shape and size of the SDV within the cell^20^.

A critical step in silicification is the concentrative uptake of silicic acid across the plasmalemma into the cell and, ultimately, to saturating concentrations within the SDV. This active transport is apparently mediated by a novel family of integral membrane proteins that function as silicic acid transporters (SITs). The SITs are found universally within the diatoms, with multiple paralogues being common^21^,^22^,^23^,^24^, and are upregulated during silicification or in response to silicic acid limitation^4^,^5^,^7^,^25^. SITs have no significant homology to any other protein sequences but hydropathy analysis suggests that they are integral membrane proteins comprising 10 transmembrane α-helices^21^. The existence of such transporters was anticipated because diatom cell cultures exhibit classical saturable transport of silicic acid under appropriate growth conditions, with *K*_M_ for silicic acid consistently within the range 0.8–8 μM (ref. 26). Because silicic acid has a p*K*a of 9.8, orthosilicic acid (Si(OH)_4_) is the dominant species except under very alkaline conditions and so is likely to be the transported form in most diatoms^27^. Saturable transport of silicic acid was reproduced in isolated diatom membrane vesicles^28^, microinjected *Xenopus laevis* oocytes^29^, and SIT proteoliposomes^30^ and was shown to depend upon the transmembrane electrochemical sodium gradient. Transport is likely to be electrogenic with 1:1 Na^+^/Si(OH)_4_ stoichiometry^28^.

In principle, the detailed structure and function of the SITs can be revealed through *in vitro* biophysical studies. In practice these studies have been challenging for several reasons. The first of these is that silicic acid is a small non-polar molecule that readily diffuses across biological membranes at high concentrations^26^. Being obliged to work at low substrate concentrations means that substrate gradients are quickly exhausted in reconstituted transport systems which have inherently poor signal-to-noise. These conditions are not compatible with the current discontinuous methods available for quantifying silicic acid (colorimetric silicomolybdate assay, inductively coupled plasma mass spectrometry and radiolabelling). Additionally, in proteoliposome experiments the silicomolybdate method cannot measure transport rates and suffers interference from phospholipids, buffer components and unreconstituted protein and is thus subject to an inherent, and substantial, variability^30^. These issues are compounded by difficulties in purifying recombinant SITs. We previously studied a SIT from *Thalassiosira pseudonana* but found this protein to be highly aggregation prone, leading to substantial batch-to-batch variation in transport assays^30^.

These challenges motivated us to perform a systematic screen for SITs that could better serve as model systems for understanding silicic acid transport. Here, we report the successful results of that screen and the ensuing protein characterization, including the development of a new fluorescent method for studying silicic acid transport kinetics.

## Results

### Expression and purification of SIT homologues

**Figure 1:**
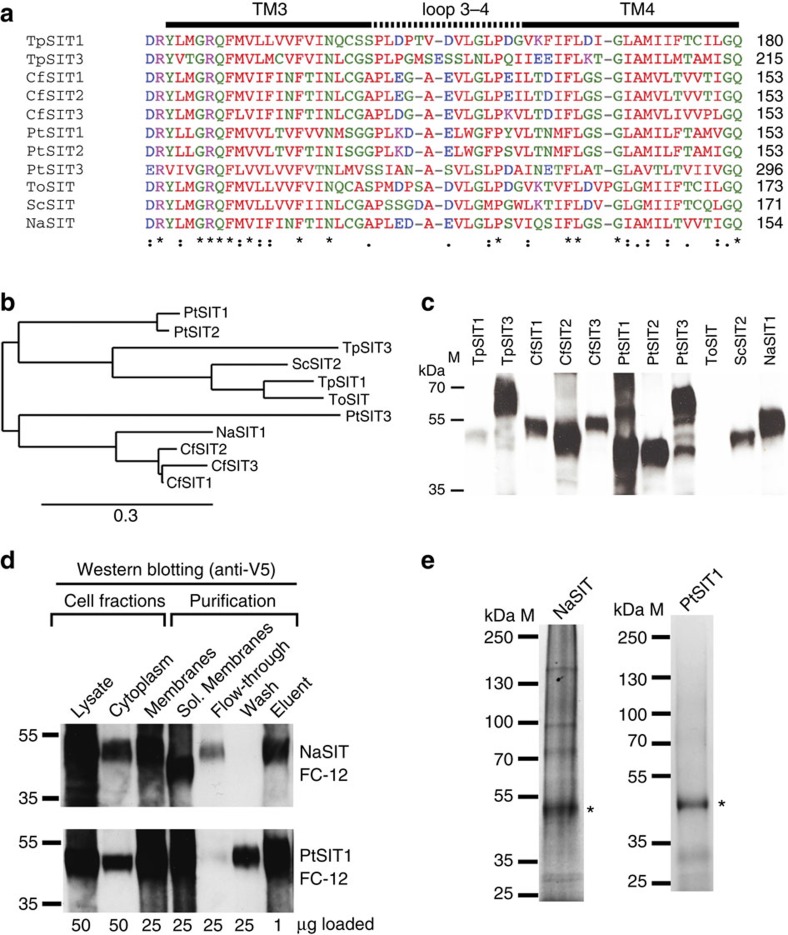
Recombinant expression of silicon transporter homologues in *S. cerevisiae*. (**a**) Representative part of a multiple sequence alignment of SIT homologues, exemplifying the results of the alignment and showing conserved transmembrane (TM) regions connected by divergent extramembrane loops. Symbols denote (*) conserved, (:) strongly similar and (.) weakly similar residues at each position. (**b**) Phylogenetic tree showing evolutionary relationships between homologues. (**c**) Qualitative western blot showing the relative expression levels of recombinant SITs in *S. cerevisiae*. Each lane contains 50 μg total protein. (**d**) Qualitative western blot tracking the fate of selected SITs during cell fractionation and affinity purification. The uncropped blot for PtSIT1 is shown in Supplementary Fig. 5. (**e**) SDS–PAGE of purified SITs stained with Coomassie Brilliant Blue. Bands corresponding to purified SITs are marked (*).

We derived a set of 11 full-length diatom SIT homologues (see Methods section). The cDNA for each of these was synthesized commercially after being codon optimized for recombinant overexpression in *Saccharomyces cerevisiae* (Supplementary Fig. 1). This set encompassed proteins from both pennate and centric diatoms including the model organisms from each of these divergent lineages, *Phaeodactylum tricornutum* and *T. pseudonana*, respectively^22^,^23^. All of these protein sequences were between 506 and 599 amino acids in length. We carried out a multiple sequence alignment of these SITs and Fig. 1a shows a representative part of this alignment that exemplifies the results. The full alignment is provided in Supplementary Fig. 2; also see Supplementary Note 1. As anticipated based on previous comparative sequence analyses^21^,^31^, the alignment revealed relatively high sequence conservation between transmembrane regions, with greater diversity in the interhelical loops and at the N- and C-termini. This included the absolute conservation of four GXQ motifs that occur in pairs at the cytoplasmic ends of helices 2 and 3 and the extracellular ends of helices 7 and 8 (Supplementary Fig. 3) and were previously suggested to be important in silicic acid binding and transport in diatoms and other silicifiers^21^,^31^. In what follows, we describe each of the SITs using conventional nomenclature with abbreviated species name and paralogue number; for example, PtSIT1 refers to paralogue 1 from *P. tricornutum.*

Initial expression trials showed that TpSIT3, CfSIT2, PtSITs1–3 and NaSIT were expressed in recombinant yeast at the highest levels (Fig. 1c). CfSIT1, CfSIT3 and ScSIT showed only moderate expression and TpSIT1 and ToSIT were expressed at very low levels. Many of the expressed proteins showed gel shifts during SDS–PAGE (polyacrylamide gel electrophoresis) to an apparent molecular weight 5–15 kDa lighter than expected from the amino acid sequence. This phenomenon is common to integral membrane proteins because of anomalous SDS loading^32^. Expressed PtSIT3 was rather heterogeneous with a ladder of lower molecular-weight bands observed that could arise from proteolysis. On this basis, and given that we had previously studied TpSIT3 (ref. 30), we decided to focus on characterizing CfSIT2, PtSIT1, PtSIT2 and NaSIT.

We next employed a high-throughput sedimentation assay^33^ to determine the solubility and dispersity (‘stability') of each of these four homologues in different detergent micelles. After treating recombinant membranes with one of 17 detergents, two consecutive ultracentrifugation runs at progressively faster speeds were used to pellet either insoluble proteins or large micellar protein aggregates. In all cases, the fos-choline series of detergents were the most effective for both solubilizing the SITs and for preventing non-specific aggregation after solubilization (Supplementary Fig. 4). This agrees with the previous results of a chromatography-based method that identified fos-choline 12 (FC-12) as being optimal for TpSIT3 (ref. 30). Based on the results of this screen, we then purified detergent-solubilized NaSIT, PtSIT1, PtSIT2 and CfSIT2 using metal affinity chromatography. Best results were achieved with PtSIT1 and NaSIT using FC-12 (Fig. 1d and Supplementary Fig. 5; also see Supplementary Note 2) with typical yields of 0.5 and 0.2 mg protein per l yeast culture for PtSIT1 and NaSIT, respectively. Purified proteins were resolved as a single band on a Coomassie-stained SDS–PAGE gel (Fig. 1e) and their identity was confirmed by mass spectrometry.

### Protein characterization

We were conscious that although some membrane proteins have been successfully studied in fos-cholines, these are notorious as strong and indiscriminate detergents that can efficiently solubilize aggregated or misfolded proteins^34^,^35^. We thus turned to biophysical methods to characterize the folding and aggregation state of solubilized SITs.

**Figure 2:**
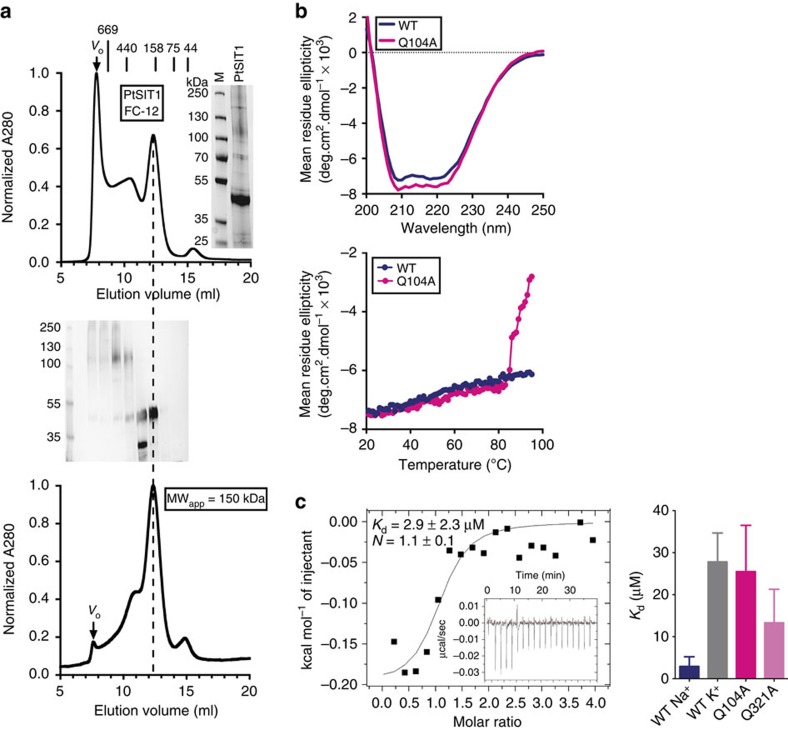
Characterization of PtSIT1. (**a**) PtSIT1 purified in FC-12 is heterogeneous during size-exclusion chromatography but a sharp peak does elute at 12.3 ml. *V*_o_, void volume. Inset, Coomassie-stained SDS–PAGE gel of loaded PtSIT1. Column fractions were analysed by SDS–PAGE with AgNO_3_ staining. The 12–13 ml fraction remains a single peak when reapplied to the column, and elutes at a volume equivalent to 150 kDa relative to molecular-weight standards as shown. (**b**) Circular dichroism spectroscopy confirms the α-helical secondary structure of PtSIT1 and a representative mutant, Q104A, and the sensitivity of these proteins to temperature. (**c**) Isothermal titration calorimetry of PtSIT1 and selected glutamine mutants. Values are derived from curve fitting to a one-site equation±s.e.m for a single experiment.

The oligomeric state of each of the purified SITs was characterized by size-exclusion chromatography (Fig. 2a). For PtSIT1 in FC-12, the chromatogram was heterogeneous and a significant fraction of the loaded protein eluted in the void volume, indicating the presence of protein aggregates above ∼600 kDa. However, we could discern a distinct peak eluting at 12.3 ml, corresponding to an apparent molecular weight of ∼150 kDa. Column fractions across the entire chromatogram were collected and visualized on an SDS–PAGE gel. The 11–12 ml fraction showed a band corresponding to PtSIT1 and another compact strong band at 30 kDa. Surprisingly, mass spectrometry revealed this low-molecular weight band to be intact full-length PtSIT1. We assume this arises from a minor state of PtSIT1 with unusual SDS binding or conformation. This low-weight band was missing in the fraction collected between 12 and 13 ml, which showed only a single major band corresponding to PtSIT1. When this fraction was reapplied to the same column it eluted as a single peak at 12.3 ml, showing that this protein population remained relatively stable in FC-12 without further aggregation. Each of the other SITs tested by size-exclusion chromatography showed substantial heterogeneity, peak broadening and aggregation (Supplementary Fig. 6).

The circular dichroism spectrum of PtSIT1 was characteristic of an α-helical protein, having strong negative deflections at 222 and 208 nm (Fig. 2b). The magnitude of those deflections was consistent with ∼25% of the protein residues being α-helical, similar to previous results with TpSIT3 (ref. 30) but slightly lower than anticipated from sequence-based structure predictions (Supplementary Fig. 3). Thermal melts showed that PtSIT1 was very stable, with no significant loss of secondary structure up to 95 °C. In contrast, mutants such as the representative Q104A had identical secondary structure at room temperature but tended to be destabilized, exhibiting a cooperative loss of secondary structure with *T*_m_ ∼80 °C.

We next used isothermal titration calorimetry (ITC) to probe substrate binding at equilibrium (Fig. 2c; Supplementary Fig. 7; Supplementary Table 1). PtSIT1 was apparently folded in FC-12 and was able to bind silicic acid. Fitting to a single-site binding equation yielded a *K*_d_ of 2.9±2.3 μM (±s.e.m.) and stoichiometry of 1.1±0.1. This binding affinity was reduced ∼10-fold in the absence of sodium with a poor fit to binding isotherms, suggesting that sodium binding is a prerequisite for high-affinity binding to silicic acid. This was supported by titration of sodium which, although giving lower quality data, did show sodium binding in the absence of silicic acid with a weaker *K*_d_ of 13.8±16.1 μM and stochiometry of 1.4±0.7. Mutations at the conserved glutamines Q104 and Q324 also reduced *K*_d_ by 10-fold and fourfold, respectively, suggesting a role for these residues in substrate binding. For wild-type PtSIT1, Δ*G* for silicic acid binding was −7.4±1.1 kcal mol^−1^ and this was reduced by ∼1 kcal mol^−1^ in the absence of sodium and for the mutants studied. Entropic changes made the major contribution to Δ*G*, with Δ*H* being only −0.2±0.03 kcal mol^−1^. This modest net enthalpy change probably excludes the formation of new hydrogen bonds and extensive van der Waals contacts between the protein and the ligand. Instead, the data suggest that silicic acid binding is characterized by favourable changes in solvent entropy arising from the desolvation of the protein or the substrate or from protein conformational shifts.

### Zinc silicate fluorescence

We next sought to develop a novel fluorescence assay that would enable us to characterize silicic acid transport. The green fluorescence of zinc silicates under UV light is well-known, being observed in the natural mineral willemite and exploited in synthetic zinc phosphors for commercial lighting, display and analytical applications^36^. Although strong fluorescence from zinc silicate requires doping with other metal ions, we reasoned that even weak fluorescence signals would be measurable by sensitive solution methods.

**Figure 3:**
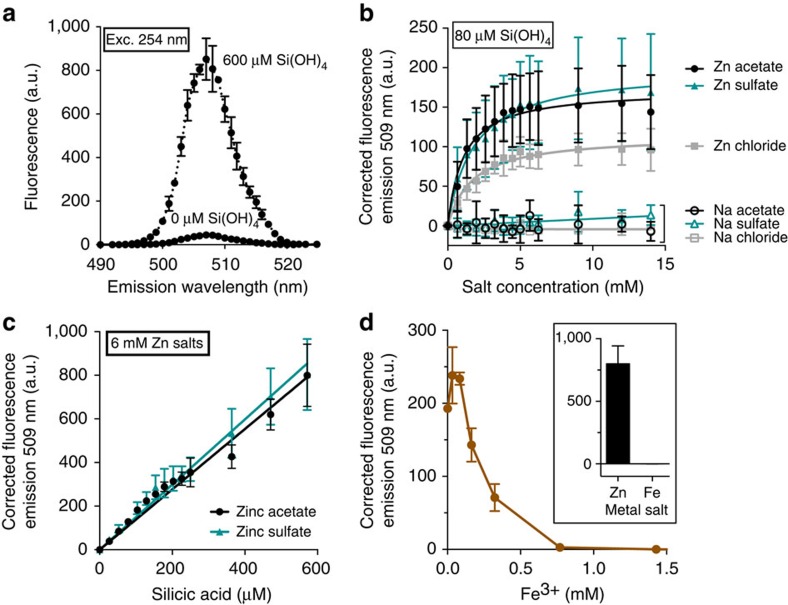
Silicic acid augments the fluorescence of Zn salts in Tris/KCl buffer. (**a**) Fluorescence emission of 6 mM Zn acetate solutions increases 20-fold in 600 μM silicic acid. (**b**) The emission signal saturates at approximately 60-fold molar excess of Zn salt. (**c**) When Zn salts are held in excess, silicic acid titration produces a linear response. (**d**) Competition with Fe^3+^ yields non-fluorescent Fe complexes. Inset, fluorescence of Zn and Fe complexes at 600 μM silicic acid. All data are mean±s.d., *n*=3.

Figure 3a shows that the presence of 600 μM silicic acid causes a 20-fold increase in the fluorescence emission of zinc acetate solutions at 507 nm. No precipitates were observed in these reactions, nor could any be detected by dynamic light scattering (DLS) measurements (Supplementary Fig. 8). We assume that the observed fluorescence comes from the formation of small Zn^2+^–polysilicate complexes below the limits of detection by DLS (≤0.4 nm).

To further investigate this phenomenon, we examined the fluorescence of 80 μM silicic acid titrated with the salts ZnSO_4_, ZnCl_2_ and Zn acetate (Fig. 3b). In each case, the fluorescence emission increased predictably until saturating at 5 mM Zn salt, or ∼60:1 molar ratio of zinc:silicic acid. The data could be fit to hyperbolic binding curves as shown, giving dissociation constants in the range 1–2 mM for each zinc salt. Zn acetate and ZnSO_4_ gave twice the emission intensity of ZnCl_2_ and so the latter was not used further. As expected, no fluorescence was observed in negative controls with the analogous sodium salts.

In the inverse experiment, Zn acetate and ZnSO_4_ were held in excess and titrated with silicic acid (Fig. 3c). In both cases the change in fluorescence emission was linear, with a gradient of 1.4±0.1 (±s.e.m.) fluorescence units μM^−1^ silicic acid. We found that titrating FeCl_3_ into Zn acetate/silicic acid mixtures abolished fluorescence (Fig. 3d), presumably because the non-fluorescent Fe^3+^ displaces Zn^2+^ according to the established silica-metal reactivity series^37^. Further experiments showed that Zn silicate fluorescence was influenced by the buffer components Tris and KCl and was essentially abolished at low pH that prevented silicic acid polymerization (Supplementary Fig. 8).

### Proteoliposome transport assays

We next used Zn silicate fluorescence to assay *in vitro* silicic acid transport by SITs. TpSIT3, NaSIT and PtSIT1 were reconstituted into proteoliposomes preloaded with 6 mM Zn acetate (Supplementary Fig. 9). An inward-directed electrochemical sodium gradient was applied to the SIT proteolipsomes and Zn silicate fluorescence from transported silicic acid was measured continuously in a stopped-flow instrument.

**Figure 4:**
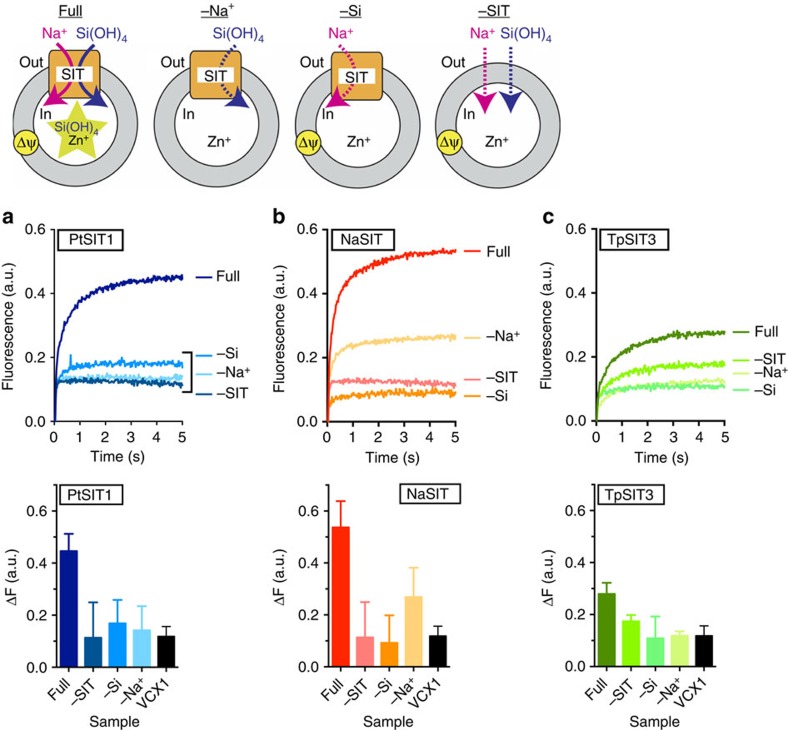
Silicic acid transport into energized Zn-loaded proteoliposomes. Averaged raw data are shown from experiments with (**a**) PtSIT1, (**b**) NaSIT and (**c**) TpSIT3. The lower panels show the mean fluorescence change±s.d. from eight technical replicates.

In the presence of 80 μM external silicic acid we observed an increase in zinc silicate fluorescence over negative controls (Fig. 4). PtSIT1 and NaSIT showed similar activity, but TpSIT3 gave a modest signal change that was only just discernible above background. As expected, the transport assay had a low signal and fast rate because of the low concentrations of silicic acid that we use to avoid artifacts from spontaneous diffusion. Control experiments consistently showed a background signal that we attribute to a liposome mixing artifact. This background was also present in an additional negative control using proteoliposomes reconstituted with an unrelated transporter, the yeast H^+^/Ca^2+^ exchanger VCX1 (ref. 38).

**Figure 5:**
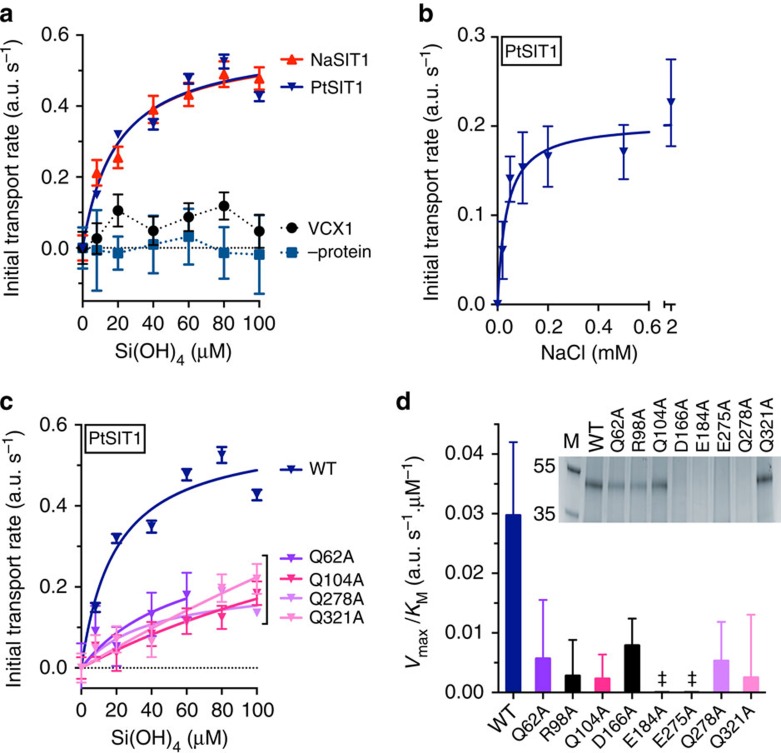
Transport kinetics of SIT proteoliposomes. (**a**) Silicic acid dependence. (**b**) Sodium dependence of PtSIT1 at 40 μM silicic acid. (**c**) Silicic acid transport is diminished by mutations at conserved glutamines as shown. For panels **a**–**c**, each datapoint is mean±s.e.m from a linear fit to the average of four technical replicates (**d**). Transport activity of WT versus mutants. (‡), not determined because data could not be fit to the Michaelis–Menten equation. Inset, Coomassie-stained SDS–PAGE gel of purified mutants. Data are mean±s.e.m from fitting to the Michaelis–Menten equation.

Given the lower activity of TpSIT3 we chose to focus on characterizing the transport kinetics of PtSIT1 and NaSIT. We determined initial (linear) transport rates at different silicic acid concentrations and constructed Michaelis–Menten plots for both homologues (Fig. 5a). Both PtSIT1 and NaSIT showed classical transport kinetics, with identical *V*_max_ of 0.6±0.1 fluorescence units s^−1^ and *K*_M_ for silicic acid of 19.1±4.5 μM and 19.6±7.7 μM for PtSIT1 and NaSIT respectively (errors are ±s.e.m from curve fitting). Control experiments showed that background diffusion of silicic acid was negligible under all conditions. Since *P. tricornutum* is a model diatom species with a sequenced genome that is readily culturable and amenable to cell biological studies^6^,^23^, we decided to concentrate our further studies on PtSIT1. We determined the sodium dependence of PtSIT1 and observed a saturable response (Fig. 5b), with 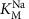 of 35.5±18.6 μM.

We next made a series of targeted alanine mutations in PtSIT1 to understand the influence of certain highly conserved residues upon protein structure and function. Our initial targets were the absolutely conserved charged residues R98 and D166, likely sited within cytoplasmic loops, and E184 and E275, which are predicted to be buried within the membrane (Supplementary Figs 2 and 3). D166 is found within the partly conserved CMLD motif in loop 4 suggested to be involved in substrate transport^39^. We also introduced independent mutations at each of the four glutamines Q62, Q104, Q278 and Q321 that are found in the context of four conserved GXQ motifs. Each of these eight mutants were expressed, purified, characterized and successfully reconstituted (Supplementary Figs 9–12). However, mutations D166A, E184A, E275A and Q278A all significantly disrupted protein folding and stability; for example, these mutants were aggregation prone and so could not be resolved on SDS–PAGE (Fig. 5d, inset). In contrast, mutations R98A, Q62A, Q104A and Q321A were relatively benign.

All of the mutants showed substantially diminished transport activity compared with wild-type PtSIT1 (Fig. 5c,d). These data are difficult to interpret for the disruptive mutants D166A, E184A, E275A and Q278A since the signal loss may arise from protein aggregation or misfolding. However, it is tempting to speculate that the reduced activity of the benign mutations Q62A, R98A, Q104A and Q321A comes from the influence of these highly conserved residues in substrate binding or another part of the transport cycle. This is reinforced by our findings that substrate binding is impaired by mutations Q104A and Q321A (Fig. 2c). This would support the hypothesis that the conserved glutamines play a role in silicic acid binding and transport and provides the first evidence that other residues, such as R98, may also be important in SIT function.

## Discussion

Biological silicon transport is an enduring enigma. How do integral membrane proteins in plants^40^,^41^,^42^, diatoms, choanoflagellates^31^ and other silicifiers recognize and transport silicic acid? Aside from the plant aquaporin-like channels, silicic acid transport appears to be an active process coupled to the dissipation of an electrochemical gradient. However, the molecular basis of the protein–silicon interaction has remained elusive. This is at least partly because of the paucity of experimental methods that have been available to study these proteins. Here, we introduce new approaches that allow us to bring *in vitro* techniques to bear upon the SITs. These enable us to characterize silicic acid binding and transport in isolation from the (very complex) cell environment and absent the dynamic regulation that occurs during the cell cycle^4^,^25^,^43^. The new methods include developing a fluorescent probe for silicic acid that expands the ‘toolkit' available for studying silicon transport *in vitro.* Using these methods, we can directly compare and contrast SITs from different diatoms and apply site-directed mutagenesis to try and identify important residues for substrate binding and transport. We anticipate that the screens of SIT expression and detergent compatibility presented here will also provide a platform for future structural biology studies that should lead to a more detailed understanding of structure and function.

We can make some general observations on diatom SITs based upon our results. The protein:silicic acid stoichiometry is 1:1 with substrate binding being entropically-driven, and both *K*_d_ and *K*_M_ are in the low micromolar range. Because we are working at near-neutral pH throughout, our results support previous reports that the undissociated orthosilicic acid is the form that interacts with the transporter^27^. Sodium is essential for active transport and is required for high-affinity substrate binding, confirming that SITs utilize sodium symport and suggesting that sodium binding precedes silicic acid binding in the transport cycle. Mutating any one of four conserved glutamines impairs substrate binding and transport, supporting the role of these residues in substrate binding.

In the case of PtSIT1, the 1:1 binding stoichiometry and hyperbolic transport kinetics observed here suggest that the SITs follow classical Michaelis–Menten kinetics *in vitro*. Although SITs expressed in *Xenopus laevis* oocytes^29^ and studied in diatom membrane vesicles^28^ also showed Michaelis–Menten kinetics, there is convincing evidence that in culture at least some diatom strains show a sigmoidal trend in silicic acid uptake which can be interpreted as arising from cooperative transport^26^. We also found such a sigmoidal trend in our own previous work^30^ studying TpSIT3 in proteoliposomes. It will thus be important for further work to clarify whether cooperative or non-cooperative transport kinetics are intrinsic properties of the SITs or instead reflect the assay system used.

**Figure 6:**
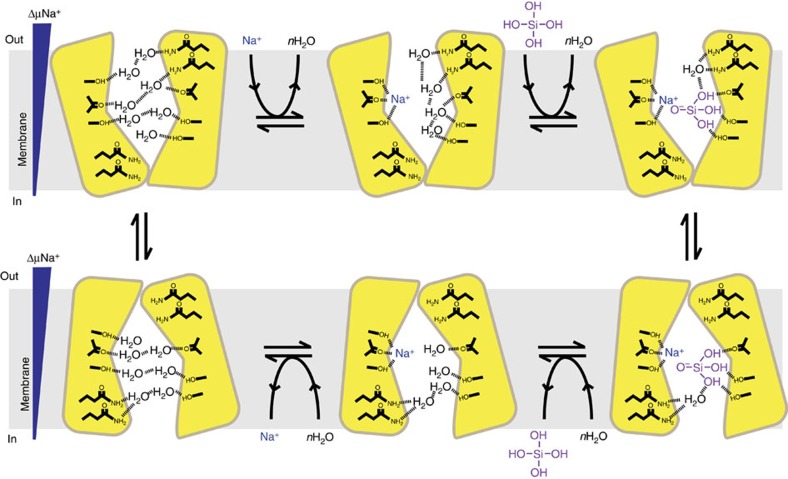
Schematic cartoon (not to scale) of an alternating access mechanism for the SIT transport cycle. Sequential co-substrate binding with 1:1 stoichiometry, driven by an increase in solvent entropy, causes a change in protein conformation that exposes the binding site to the opposite side of the membrane. After substrate dissociation, the empty SIT then returns to the original conformation. Protein–substrate interactions could be mediated by either side chain or main chain groups.

Our results cannot easily be explained by a current model^9^,^21^ which suggests that the SITs contain two functionally equivalent binding sites at opposing sides of the membrane. Instead, we propose that our results support an alternative mechanism shown in Fig. 6 and discussed below. Although our model is consistent with the experimental data presented here and elsewhere, it is necessarily speculative and should be viewed as such.

The overarching principle of our model is that SITs operate via an alternating access mechanism. Some variation upon this mechanism is universally observed for solute transporters^44^,^45^,^46^ because it allows the substrate to be moved from one side of the bilayer to the other without compromising bilayer integrity. In keeping with other transporters, our model supposes that the SIT has a substrate binding site at the centre of the membrane; and that protein conformational changes, driven by the co-ordinated binding and release of substrates, alternately expose this binding site to the opposing faces of the bilayer without ever opening a continuous pore.

Given the 1:1:1 Na^+^:Si(OH)_4_:SIT stoichiometry suggested by ITC (Fig. 2; Supplementary Fig. 7; Supplementary Table 1) we suggest that there is a single shared binding site for sodium and silicic acid at the centre of the membrane. Further, ITC also shows that silicic acid cannot readily bind in the absence of sodium, but that sodium can bind in the absence of silicic acid. Because of this, we suggest that sodium binding to an outward-facing conformation occurs as a first step in the transport cycle and predisposes the transporter to bind silicic acid. Sodium-binding sites from other transporters consist of multiple interactions between Na^+^ and polar amino acid side chains, as well as interactions with main chain carbonyls^44^,^47^. We assume that the sodium-binding site in the SITs will be similar. Candidates for polar side chains involved in sodium binding include S20, Q104, N115, H190, Y193, S229 and S372 (numbers refer to PtSIT1 sequence) based on their absolute conservation in our multiple sequence alignment (Supplementary Fig. 2).

The binding of sodium then facilitates the binding of silicic acid. We propose that sodium and silicic acid come in close proximity within the binding site. Although monomeric silicic acid does not readily interact with sodium in solution^37^, we hypothesize that the close approach of the two cosubstrates within the microenvironment of the binding site induces the ionization of silicic acid to Si(OH)_3_O^−^ and establishes an electrostatic interaction between Si(OH)_3_O^−^ and Na^+^. This induced dipole allows the remaining silanols to engage in hydrogen bonding with amino acid side chains or main chain carbonyls. It is well-established that silanol groups can hydrogen bond with peptides^48^ and other organic polymers^37^,^49^ and silicic acid probably makes up to four hydrogen bonds with water^37^. These protein-silanol bonds displace protein-water hydrogen bonds and so disrupt the water network within the binding site. This causes an increase in solvent entropy, consistent with our results that suggest entropy is the major driving force for silicic acid binding. Such a mechanism requires that multiple waters are displaced by a single silicic acid and this is shown in Fig. 6. The overall binding entropy determined by experiment is the sum of individual contributions from the protein, the ligand and the solvent^50^,^51^. Resolving the contribution of each of these terms to the total entropy is difficult, but for the binding of a relatively simple small molecule like silicic acid changes in ligand and protein conformational entropy are likely to be negligible. We thus suggest that solvent entropy from protein and/or ligand desolvation makes the largest contribution. Our proposed mechanism may also resolve the quandary of the conserved glutamines, which are implicated in substrate binding (Figs 2 and 5) but cannot be part of a central binding site since they are situated at either side of the bilayer close to the headgroup region. We suggest that although they may not contact the substrate directly, these glutamines might have an important indirect role in organizing the water network. We have given them this role in Fig. 6.

The next step in the transport cycle is a change in protein conformation that exposes the binding site to the cell interior. After this conformational change the first half of the cycle is reversed, so that water enters the binding site and replaces Si(OH)_3_O^−^, which is immediately reprotonated to Si(OH)_4_. Sodium then leaves and the empty SIT resets to the outward-facing conformation.

To our knowledge, the SITs are the only proteins yet known that can specifically recognize silicic acid with high affinity. There has been keen interest in studying proteins and peptides that can interact with inorganic materials and with soluble mineral precursors^52^ because understanding the molecular basis of such interactions allows them to be translated into biomimetic materials chemistry^53^,^54^. A general advantage of biomimetic chemistry is that it can take place under mild conditions within the physiological range of pH and temperature. This has prompted suggestions that bioinspired methods could be an environmentally friendly alternative to existing manufacturing processes for silica^55^. Thus understanding the specific and unusual protein-silicic acid interface found in the SITs may provide a fresh impetus to the biomimetic ‘green' synthesis of silica materials.

Diatoms play a major role in marine biology and global biogeochemistry^2^,^3^,^56^,^57^,^58^. Silicic acid, which is present at low micromolar concentrations in much of the surface ocean, is an essential and limiting nutrient for the growth of many diatoms and cellular demand can only be met via concentrative active transport by the SITs^59^,^60^. Understanding silicic acid transport and utilization thus offers key insights into diatom growth cycles, community dynamics and competition for resources^61^,^62^. Information on silicic acid transport can also supply the parameters for computational models of the marine ecosystem^63^,^64^, providing a link between diatom cellular biology and biogeochemical cycles at the global scale.

One outstanding question is whether different SITs will have different transport characteristics. Studies of cell cultures suggest that the rate and efficiency of silicic acid transport vary between diatom species and that SITs from marine diatoms may have a higher affinity for silicic acid than those from freshwater diatoms^60^. These variations probably represent differences in SIT expression levels and regulation, as well as physiological diversity and environmental pressures, rather than the evolution of fundamentally different transport mechanisms^65^. Additionally, because diatoms often contain multiple SIT paralogues, there may be lower- and higher-affinity SIT variants within the same cell that predominate at different stages of silicification^24^,^60^. Although we cannot address these issues definitively, our results suggest that homologues PtSIT1 and NaSIT from different marine species are functionally indistinguishable (Fig. 5). In particular, we find that PtSIT1 has a *K*_M_ for sodium in the micromolar range. This seems unnecessarily stringent in *P. tricornutum*, a coastal marine diatom that constantly experiences millimolar salt gradients across the cell membrane. However, relatively high sodium affinities would allow the SITs to be active as sodium symporters in freshwater diatoms where salt gradients might be shallower or more variable; clearly this could be confirmed by studying SITs from freshwater diatoms. In this context it is also interesting to consider the functional diversity of silicon transport proteins from different organisms. The choanoflagellate SITs are evolutionarily related to the diatom SITs and so are probably also sodium-driven symporters^31^. In sponges, there is circumstantial evidence that a protein belonging to the Na^+^/HCO_3_^−^ transporter family may be involved in silicic acid transport^66^. In the case of plants, silicon transporters are either aquaporin-like channels^40^,^67^ or appear to be proton-driven secondary symporters^41^. Thus cation symport appears to be a common mechanism among those SITs that function as active secondary transporters.

We thus take a step towards revealing the detailed mechanism of silicon transport in the diatom. This will ultimately help to realize the exploitation of diatoms in biotechnology, provide an inspiration for new composite materials, and support the pressing need to understand global biogeochemistry.

## Methods

### Identification and synthesis of SIT homologues

Interrogating the NCBI protein database (http://www.ncbi.nlm.nih.gov) with the search term ‘silicon transporter AND stramenopiles[orgn]' in November 2013 yielded 28 full-length sequences for diatom silicon transporters. Of these, one sequence from *Thalassiosira oceanica* was unusually large (761 amino acids) and was excluded as being non-representative. A multiple sequence alignment of the remaining 27 sequences using Clustal Omega (http://www.ebi.ac.uk/Tools/msa/clustalo/)^68^ revealed that several of these proteins were found to be either redundant depositions or highly similar, with sequence identities >90%. In this case, one of these virtually identical proteins was arbitrarily selected for further study. This ultimately provided a set of 11 full-length SITs. The transmembrane topology of PtSIT1 was predicted by the online tools TMPred (http://www.ch.embnet.org/software/TMPRED_form.html), HMMTOP (http://www.enzim.hu/hmmtop/), TMHMM (http://www.cbs.dtu.dk/services/TMHMM/) and TOPCONS (http://topcons.cbr.su.se). These were in reasonable agreement except that Helix 8 was only weakly predicted by TMMHMM. The overall consensus of the topology predictions was used to generate a schematic topology diagram with TOPO2 (http://www.sacs.ucsf.edu/TOPO2/).

Each of these constructs was made as a synthetic gene by Eurofins Genomics or DNA 2.0 (Supplementary Fig. 1). The synthetic genes were codon optimized for expression in *S. cerevisiae* either using online tools (http://www.jcat.de) or with the manufacturers proprietary software. Synthetic genes were subcloned into the expression plasmid pYES2 (Life Technologies) with the stop codon omitted to allow read-through into a V5 epitope and polyhistidine tag. Plasmids were transformed into the protease-deficient *S. cerevisaie* strain FGY217 (*MAT*α*, ura3-52, lys2*Δ*201, pep4*Δ)^69^. Site-directed mutagenesis of these constructs was via the plasmid amplification method.

### Protein expression

Small-scale expression tests were carried out in 10 ml of auxotrophic yeast media (-Ura). Cultures grown at 30 °C to an OD_600_ of 0.4 were induced with 2% (w/v) galactose for 24 h. Cell lysates were boiled at 95 °C for 10 min in sample application buffer and 50 μg of total lysate protein was loaded onto 12% Tris-Glycine SDS–PAGE gels. SIT expression levels were detected by western blotting with anti-V5-HRP at 1:10,000 dilution (Life Technologies, catalogue number R96125).

### Sedimentation dispersity assay

Recombinant yeast membranes were treated with detergent and subject to two consecutive ultracentrifugation runs at 140,000*g* and 300,000*g* to pellet unsolubilized proteins and large protein aggregates, respectively. The supernatant from each run was collected and the protein qualitatively assessed by western blotting.

### Protein purification and characterization

For purification, primary cultures were harvested at 3,500*g* and added to a final OD600 of 0.4 in 1 l secondary cultures containing 2% galactose and 0.1% glucose. After 24 h at 30 °C, cells were harvested at 3,500*g* and resuspended in 100 ml phosphate buffered saline (1 × PBS). Cells were lysed in a continuous flow cell disrupter (Constant Systems Ltd) at 35 KPSI. After a clearing spin at 10,000*g* for 10 min, cell membranes were sedimented at 180,000*g* for 1 h. Membrane were resuspended in 50 mM TrisHCl, pH 7.4, 150 mM NaCl, 5% Glycerol and homogenized. The total protein was determined by the detergent-compatible Lowry assay (DC Protein Assay, Bio-Rad Laboratories Inc.) and the membrane suspension was diluted to 5 mg ml^−1^. Detergents were then added to at least 10 × above the critical micelle concentration for solubilization. After 1 h at room temperature, insoluble material was pelleted at 180,000*g* for 1 h. The soluble supernatant was retained and imidazole added to 20 mM before being applied to a 1 ml ‘HisTrap' Ni^2+^ column (GE Healthcare) equilibrated in Column Buffer (50 mM TrisHCl, pH 7.4, 150 mM NaCl, 5% glycerol, detergent at least 4x critical micelle concentration) plus 20 mM imidazole. Specifically, FC-12 was used at 2% for membrane solubilization and 0.1% thereafter. After washing with 40 column volumes of Column Buffer plus 60 mM imidazole, SITs were eluted in the same buffer with 0.5 M imidazole which was immediately removed by gel filtration.

Size-exclusion chromatography was performed with a prepacked Superdex S200 10/300 GL column (GE Healthcare) at a flow rate of 0.5 ml min^−1^. Circular dichroism spectroscopy was performed on a Jasco J-1500 instrument with a 1 mm pathlength cell at protein concentrations of 0.2–1 mg ml^−1^. Thermal melts were recorded at 1 °C steps with 10 s equilibration time. Isothermal titration calorimetry was carried out on a MicroCal iTC-200 at protein concentrations of 30 μM (2 mg ml^−1^), giving *c*-values ∼10, and ligand concentration of 400–800 μM. Injections were 18 × 2 μl at 120 s intervals. Data were fit to a single-site binding equation using the instrument software.

### Protein reconstitution

Liposomes were prepared from *E. coli* polar lipid mix and egg phosphatidylcholine (Avanti Polar Lipids) dissolved at 3:1 w/w respectively in 1:1 chloroform:methanol and dried to a lipid film. The mixed lipids were resuspended at 40 mg ml^−1^ in 50 mM Tris, 150 mM KCl, pH 7.4 and sonicated in a bath-type sonicator for 15 min before being subject to five freeze/thaw cycles using liquid nitrogen and a waterbath set at 42 °C. Liposomes were formed by extruding the lipid suspension through a 0.4 μm filter in a bench-top extruder (Avanti Polar Lipids) according to the manufacturers' instructions at room temperature. For reconstitution, 500 μl extruded liposomes were incubated with 45 μl of 20% sodium cholate and 55 μl SIT at 360:1 (w/w) lipid:protein ratio. After 30 min at room temperature cholate was removed by gel filtration with an equilibrated PD SpinTrap G-25 column using the centrifugation rather than gravity method. The eluted proteoliposomes were centrifuged at 200,000*g* for 1 h. For influx assays, the pellet was resuspended at 40 mg ml^−1^ in 50 mM Tris, 150 mM KCl, pH 7.4. The buffer composition was adjusted as required for control experiments.

For sucrose flotation assays, samples were diluted into 60% sucrose and loaded onto a discontinuous sucrose gradient comprising 1 volume of sample in 60% sucrose, 4.5 volumes of 40% sucrose and 1 volume of buffer. Gradients were centrifuged at 180,000*g* for 1 h and fractions removed by syringe needle for western blotting.

### Zinc acetate fluorescence assay

Silicic acid was either prepared by incubating 4 ml of 0.2 M sodium silicate with 1.5 g of acidified Dowex 50WX4–50 cation exchange resin or by dilution of sodium silicate, and silicic acid concentration was verified by silicomolybdate assays and ICP-MS. For kinetic influx measurements, proteoliposomes in 50 mM Tris, 150 mM KCl, pH 7.4 were loaded with 6 mM Zn acetate by two cycles of freeze-thaw and treated with 1μM valinomycin. A stopped-flow instrument (Applied Photophysics) was configured so that each reaction comprised one volume proteoliposomes and ten volumes of 50 mM Tris, 150 mM NaCl, pH 7.4 plus silicic acid as required. This instantaneously establishes an electrochemical sodium gradient (150 mV, negative inside), with the electrical component generated by the egress of potassium ions through the valinomycin pore. Fluorescence excitation was at 254 nm with a 360 nm cut-off filter. Initial rates were determined from the early part of the kinetic trace and fit to the Michaelis–Menten equation by non-linear curve-fitting in GraphPad Prism.

### Data availability

The authors declare that the data supporting the findings of this study are available from the corresponding author on reasonable request.

## Additional information

**How to cite this article:** Knight, M. J. *et al.* Direct evidence of the molecular basis for biological silicon transport. *Nat. Commun.* 7:11926 doi: 10.1038/ncomms11926 (2016).

## Supplementary Material

Supplementary Information Supplementary Figures 1-12, Supplementary Tables 1, Supplementary Notes 1-2 and Supplementary References.

## References

[b1] Round F. E., Crawford R. M. & Mann D. G. The Diatoms: biology and Morphology of the Genera Cambridge University Press (1990).

[b2] Armbrust E. V. The life of diatoms in the world's oceans. Nature 459, 185–192 (2009). 19444204 10.1038/nature08057

[b3] Field C. B., Behrenfeld M. J., Randerson J. T. & Falkowski P. Primary production of the biosphere: integrating terrestrial and oceanic components. Science 281, 237–240 (1998). 9657713 10.1126/science.281.5374.237

[b4] Shrestha R. P. *et al.* Whole transcriptome analysis of the silicon response of the diatom *Thalassiosira pseudonana*. BMC Genomics 13, 499 (2012). 22994549 10.1186/1471-2164-13-499 PMC3478156

[b5] Mock T. *et al.* Whole-genome expression profiling of the marine diatom *Thalassiosira pseudonana* identifies genes involved in silicon biogenesis. Proc. Natl Acad. Sci. USA 105, 1579–1584 (2008). 18212125 10.1073/pnas.0707946105 PMC2234187

[b6] Sapriel G. *et al.* Genome-wide transcriptome analyses of silicon metabolsim in *Phaeodactylum tricornutum* reveal the multilevel regulation of silicic acid transporters. PLoS ONE 4, e7458 (2009). 19829693 10.1371/journal.pone.0007458 PMC2758714

[b7] Hildebrand M., Higgins D. R., Busser K. & Volcani B. E. Silicon-responsive cDNA clones isolated from the marine diatom *Cylindrotheca fusiformis*. Gene 132, 213–218 (1993). 8224866 10.1016/0378-1119(93)90198-c

[b8] Kröger N. & Poulsen N. Diatoms: from cell wall biogenesis to nanotechnology. Annu. Rev. Genet. 42, 83–107 (2008). 18983255 10.1146/annurev.genet.41.110306.130109

[b9] Hildebrand M. Diatoms, biomineralization processes, and genomics. Chem. Rev. 108, 4855–4874 (2008). 18937513 10.1021/cr078253z

[b10] Kröger N., Deutzmann R., Bergsdorf C. & Sumper M. Species-specific polyamines from diatoms control silica morphology. Proc. Natl Acad. Sci. USA 97, 14133–14138 (2000). 11106386 10.1073/pnas.260496497 PMC18883

[b11] Kröger N., Deutzmann R. & Sumper M. Polycationic peptides from diatom biosilica that direct silica nanosphere formation. Science 286, 1129–1132 (1999). 10550045 10.1126/science.286.5442.1129

[b12] Kröger N., Deutzmann R. & Sumper M. Silica precipitating peptides from diatoms. The chemical structure of silaffin-A from *Cylindrotheca fusiformis*. J. Biol. Chem. 276, 26066–26070 (2001). 11349130 10.1074/jbc.M102093200

[b13] Kröger N., Lorenz S., Brunner E. & Sumper M. Self-assembly of highly phosphorylated silaffins and their function in biosilica morphogenesis. Science 298, 584–586 (2002). 12386330 10.1126/science.1076221

[b14] Wenzl S., Hett R., Richthammer P. & Sumper M. Silacidins: Highly acidic phosphoeptides from diatom shells assist in silica precipitation *in vitro*. Angew. Chem. Int. Ed. Engl. 47, 1729–1732 (2008). 18203228 10.1002/anie.200704994

[b15] Kröger N., Bergsdorf C. & Sumper M. Frustulins: domain conservation in a protein family associated with diatom cell walls. Eur. J. Biochem. 239, 259–264 (1996). 8706728 10.1111/j.1432-1033.1996.0259u.x

[b16] Kröger N., Lehmann G., Rachel R. & Sumper M. Characterization of a 200-kDa diatom protein that is specifically associated with a silica-based substructure of the cell wall. Eur. J. Biochem. 250, 99–105 (1997). 9431996 10.1111/j.1432-1033.1997.00099.x

[b17] Scheffel A., Poulsen N., Shian S. & Kröger N. Nanopatterned protein microrings from a diatom that direct silica morphogenesis. Proc. Natl Acad. Sci. USA 108, 3175–3180 (2011). 21300899 10.1073/pnas.1012842108 PMC3044418

[b18] Tesson B. & Hildebrand M. Characterization and localization of insoluble organic matrices associated with diatom cell walls: insight into their roles during cell wall formation. PLoS ONE 8, e61675 (2013). 23626714 10.1371/journal.pone.0061675 PMC3633991

[b19] Buhmann M. T. *et al.* A tyrosine-rich cell surface protein in the diatom *Amphora coffeaeformis* identified through transcriptome analysis and genetic transformation. PLoS ONE 9, e110369 (2014). 25372470 10.1371/journal.pone.0110369 PMC4220933

[b20] Tesson B. & Hildebrand M. Extensive and intimate association of the cytoskeleton with forming silica in diatoms: control over patterning on the meso- and micro-scale. PLoS ONE 5, e14300 (2010). 21200414 10.1371/journal.pone.0014300 PMC3000822

[b21] Thamatrakoln K., Alverson A. J. & Hildebrand M. Comparative sequence analysis of diatom silicon transporters: towards a mechanistic model of silicon transport. J. Phycol. 42, 822–834 (2006).

[b22] Armbrust E. V. *et al.* The genome of the diatom Thalassiosira pseudonana: ecology, evolution and metabolism. Science 306, 79–86 (2004). 15459382 10.1126/science.1101156

[b23] Bowler C. *et al.* The *Phaeodactylum* genome reveals the evolutionary history of diatom genomes. Nature 456, 239–244 (2008). 18923393 10.1038/nature07410

[b24] Hildebrand M., Dahlin K. & Volcani B. E. Characterization of a silicon transporter gene family in *Cylindrotheca fusiformis*: sequences, expression analysis, and identification of homologs in other diatoms. Mol. Gen. Genet. 260, 480–486 (1998). 9894919 10.1007/s004380050920

[b25] Shrestha R. P. & Hildebrand M. Evidence for a regulatory role of diatom silicon transporters in cellular silicon responses. Eukaryot. Cell 14, 29–40 (2015). 25380754 10.1128/EC.00209-14 PMC4279021

[b26] Thamatrakoln K. & Hildebrand M. Silicon uptake in diatoms revisited: a model for saturable and non-saturable uptake kinetics and the role of silicon transporters. Plant Physiol. 146, 1397–1407 (2008). 18162598 10.1104/pp.107.107094 PMC2259041

[b27] Del Amo Y. & Brzezinski M. A. The chemical form of dissolved Si taken up by marine diatoms. J. Phycol. 35, 1162–1170 (1999).

[b28] Bhattacharya P. & Volcani B. E. Sodium-dependent silicate transport in the apochlorotic marine diatom *Nitzschia alba*. Proc. Natl Acad. Sci. USA 77, 6386–6390 (1980). 16592914 10.1073/pnas.77.11.6386 PMC350289

[b29] Hildebrand M., Volcani B. E., Gassmann W. & Schroeder J. I. A gene family of silicon transporters. Nature 385, 688–689 (1997). 9034185 10.1038/385688b0

[b30] Curnow P. *et al.* Expression, purification and reconstitution of a diatom silicon transporter. Biochemistry 51, 3776–3785 (2012). 22530967 10.1021/bi3000484

[b31] Marron A. O. *et al.* A family of diatom-like silicon transporters in the siliceous loricate choanoflagellates. Proc. Biol. Sci. 280, 20122543 (2013). 23407828 10.1098/rspb.2012.2543 PMC3574361

[b32] Rath A., Glibowicka M., Nadeau V. G., Chen G. & Deber C. M. Detergent binding explains anomalous SDS-PAGE migration of membrane proteins. Proc. Natl Acad. Sci. USA 106, 1760–1765 (2009). 19181854 10.1073/pnas.0813167106 PMC2644111

[b33] Gutman D. A. P. *et al.* A high-throughput method for membrane protein solubility screening: the ultracentrifugation dispersity sedimentation assay. Protein Sci. 16, 1422–1428 (2007). 17567744 10.1110/ps.072759907 PMC2206705

[b34] Thomas J. A. & Tate C. G. Quality control in eukaryotic membrane protein overproduction. J. Mol. Biol. 426, 4139–4154 (2014). 25454020 10.1016/j.jmb.2014.10.012 PMC4271737

[b35] Newstead S., Kim H., von Heijne G., Iwata S. & Drew D. High-throughput fluorescent-based optimization of eukaryotic membrane protein overexpression in *Saccharomyces cerevisiae*. Proc. Natl Acad. Sci. USA 104, 13936–13941 (2007). 17709746 10.1073/pnas.0704546104 PMC1955786

[b36] Takesue M., Hayashi H. & Smith R. L. Jr Thermal and chemical methods for producing zinc silicate (willemite): a review. Prog. Cryst. Growth Charact. Mater. 55, 98–124 (2009).

[b37] Iler R. K. The Chemistry of Silica John Wiley & Sons, Inc (1979).

[b38] Waight A. B. *et al.* Structural basis for alternating access of a eukaryotic calcium/proton exchanger. Nature 499, 107–110 (2013). 23685453 10.1038/nature12233 PMC3702627

[b39] Shcherbakova T. A. *et al.* Conservative motif CMLD in silicic acid trasport proteins of diatom algae. Mol. Biol. (Mosk) 39, 303–316 (2005). 15856954

[b40] Ma J. F. *et al.* A silicon transporter in rice. Nature 440, 688–691 (2006). 16572174 10.1038/nature04590

[b41] Ma J. F. *et al.* An efflux transporter of silicon in rice. Nature 448, 209–212 (2007). 17625566 10.1038/nature05964

[b42] Ma J. F. & Yamaji N. Functions and transport of silicon in plants. Cell. Mol. Life Sci. 65, 3049–3057 (2008). 18560761 10.1007/s00018-008-7580-x PMC11131740

[b43] Thamatrakoln K. & Hildebrand M. Analysis of *Thalassiosira pseudonana* silicon transporters indicates distinct regulatory levels and transport activity through the cell cycle. Eukaryot. Cell 6, 271–279 (2007). 17172435 10.1128/EC.00235-06 PMC1797941

[b44] Krishnamurthy H., Piscitelli C. L. & Gouaux E. Unlocking the molecular secrets of sodium-coupled transporters. Nature 459, 347–355 (2009). 19458710 10.1038/nature08143 PMC6821466

[b45] Slotboom D. J. Structural and mechanistic insights into prokaryotic energy-coupling factor transporters. Nat. Rev. Microbiol. 12, 79–87 (2014). 24362466 10.1038/nrmicro3175

[b46] Shi Y. Common folds and transport mechanisms of secondary active transporters. Annu. Rev. Biophys. 42, 51–72 (2013). 23654302 10.1146/annurev-biophys-083012-130429

[b47] Khafizov K. *et al.* Investigation of the sodium-binding sites in the sodium-coupled betaine transporter BetP. Proc. Natl Acad. Sci. USA 109, E3035–E3044 (2012). 23047697 10.1073/pnas.1209039109 PMC3497817

[b48] Patwardhan S. V. *et al.* Chemistry of aqueous silica nanoparticle surfaces and the mechanism of selective peptide adsorption. J. Am. Chem. Soc. 134, 6244–6256 (2012). 22435500 10.1021/ja211307u

[b49] Prearl M., Spinde K., Lazic J., Brunner E. & Demadis K. D. Bioinspired insights into silicic acid stabilization mechanisms: the dominant role of polyethylene glycol-induced hydrogen bonding. J. Am. Chem. Soc. 136, 4236–4244 (2014). 24564240 10.1021/ja411822s

[b50] Leavitt S. & Freire E. Direct measurement of protein binding energetics by isothermal titration calorimetry. Curr. Opin. Struct. Biol. 11, 560–566 (2001). 11785756 10.1016/s0959-440x(00)00248-7

[b51] Frederick K. K., Marlow M. S., Valentine K. G. & Wand J. A. Conformational entropy in molecular recognition by proteins. Nature 448, 325–329 (2007). 17637663 10.1038/nature05959 PMC4156320

[b52] Chen C. L. & Rosi N. L. Peptide-based methods for the preparation of nanostructured inorganic materials. Angew. Chem. Int. Ed. Engl. 49, 1924–1942 (2010). 20183835 10.1002/anie.200903572

[b53] Nudelman F. & Sommerdijk N. A. J. M. Biomineralization as an inspiration for materials chemistry. Angew. Chem. Int. Ed. Engl. 51, 6582–6596 (2012). 22639420 10.1002/anie.201106715

[b54] Sarikaya M., Temerler C., Jen A. K.-Y., Schulten K. & Baneyx F. Molecular biomimetics: nanotechnology through biology. Nat. Mater. 2, 577–585 (2003). 12951599 10.1038/nmat964

[b55] Drummond C., McCann R. & Patwardhan S. V. A feasibility study of the biologically inspired green manufacturing of precipitated silica. Chem. Eng. J. 244, 483–492 (2014).

[b56] Nelson D. M., Treguer P., Brzezinski M. A., Leynaert A. & Quéguiner B. Production and dissolution of biogenic silica in the ocean: revised global estimates, comparison with regional data and relationship to biogenic sedimentation. Global Biogeochem. Cy. 9, 359–372 (1995).

[b57] Treguer P. J. & De La Rocha C. L. The world ocean silica cycle. Annu. Rev. Mar. Sci. 5, 477–501 (2013). 10.1146/annurev-marine-121211-172346 22809182

[b58] Bowler C., Vardi A. & Allen A. E. Oceanographic and biogeochemical insights from diatom genomes. Annu. Rev. Mar. Sci. 2, 333–365 (2010). 10.1146/annurev-marine-120308-081051 21141668

[b59] Thamatrakoln K. & Kustka A. B. When to say when: can excessive drinking explain silicon uptake in diatoms? Bioessays 31, 322–327 (2009). 19260019 10.1002/bies.200800185

[b60] Martin-Jezequel V., Hildebrand M. & Brzezinski M. A. Silicon metabolism in diatoms: implications for growth. J. Phycol. 36, 821–840 (2000).

[b61] Assmy P. *et al.* Thick-shelled, grazer-protected diatoms decouple ocean carbon and silicon cycles in the iron-limited Antarctic Circumpolar Current. Proc. Natl Acad. Sci. USA 110, 20633–20638 (2013). 24248337 10.1073/pnas.1309345110 PMC3870680

[b62] Brzezinski M. Cell-cycle effects on the kinetics of silicic acid uptake and resource competition among diatoms. J. Plankton Res. 14, 1511–1539 (1992).

[b63] Follows M. J., Dutkiewicz S., Grant S. & Chisholm S. W. Emergent biogeography of microbial communities in a model ocean. Science 315, 1843–1846 (2007). 17395828 10.1126/science.1138544

[b64] Worden A. Z. *et al.* Rethinking the marine carbon cycle: factoring in the multifarious lifestyles of microbes. Science 347, 735 (2015). 10.1126/science.1257594 25678667

[b65] Alverson A. J. Strong purifying selecton in the silicon transporters of marine and freshwater diatoms. Limnol. Oceanogr. 52, 1420–1429 (2007).

[b66] Schröder H.-C. *et al.* Silica transport in the demosponge *Suberites domuncula*: fluorescence emission analysis using the PDMPO probe and cloning of a potential transporter. Biochem. J. 381, 665–673 (2004). 15128286 10.1042/BJ20040463 PMC1133875

[b67] Trembath-Reichert E., Wilson J. P., McGlynn S. E. & Fisher W. W. Four hundred million years of silica biomineralization in land plants. Proc. Natl Acad. Sci. USA 112, 5449–5454 (2015). 25825729 10.1073/pnas.1500289112 PMC4418875

[b68] Sievers F. *et al.* Fast, scalable generation of high-quality protein multiple sequence alignments using Clustal Omega. Mol. Syst. Biol. 7, 539 (2011). 21988835 10.1038/msb.2011.75 PMC3261699

[b69] Kota J., Gilstring C. F. & Ljungdahl P. O. Membrane chaperone Shr3 assists in folding amino acid permeases preventing precocious ERAD. J. Cell Biol. 176, 617–628 (2007). 17325204 10.1083/jcb.200612100 PMC2064020

