# Supplementary material for: Direct evidence of the molecular basis for biological silicon transport
Source: Nat Commun. 2016 Jun 16;7:11926. doi: 10.1038/ncomms11926 (PMC4912633; doi:10.1038/ncomms11926)
Supplement: Supplementary Information — Supplementary Figures 1-12, Supplementary Tables 1, Supplementary Notes 1-2 and Supplementary References. [file ncomms11926-s1.pdf]

1. CODON-OPTIMIZED SIT SEQUENCES

```
>T_pseudonana_SIT1co
ATGTCTACTGCTGAAATCCAATCTGGTGCTGACACTGCTCCAGTTAAGCA      50
CGACGACCACGCTGACTCTCACGACGTTAAGTTGACTCCATTCAACATCT      100
TGAGATACATCGGTTCTATCGGTTTGTTGATCTTCTCTATCATCATCGTT      150
GGTGCTTTGATGTTCACTGGTAACACTAGAGTTGCTAAGGACGCTAACCC      200
ATGGGTTTCTTTGATCGTTTGTATCTTGGCTATCGTTTGGTTGTCTATGA      250
TCGAAGGTCAACAAGCTTCTTTGGTTGGTTTGCCACCAGTTGACCCAGAC      300
TTGTACAAGGACTCTCACCCATTGACTTACAAGAACGCTGCTTTGGCTTT      350
CAAGGGTGACAACTTGGACAGATACTTGATGGGTAGACAATTCATGGTTT      400
TGTTGGTTGTTTTCGTTATCAACCAATGTTCTTCTCCATTGGACCCAACT      450
GTTGACGTTTTGGGTTTGCCAGACGGTGTTAAGTTCATCTTCTTGGACAT      500
CGGTTTGGCTATGATCATCTTCACTTGTATCTTGGGTCAATTGACTACTC      550
AAGTTAACGCTTCTTACGCTATGATCGACTTCATCAACAACTACTTCGCT      600
TTGTTCACTTTGTACACTACTATGGCTGTTGAATTCTCTGGTATCATGCA      650
CTCTTCTTACTTGATCCAAAACATCTTGTCTGCTGTTTCTGGTAAGCCAA      700
TCCAAACTAACGAAGAACCAAAGACTGGTATGACTTTCGCTTTCTTCTGG      750
GGTAGAGTTTTGATGTCTTTGGCTATCTTGGGTTTCTGTTTGGCTGTTAC      800
TTTGGTTGCTTTGTTCAACGGTCAAACTTCTGTTTCTGTTAAGTACCCAT      850
CTATCTCTCCAGGTTTGTCTGTTTTCTTGTTGTTCTTCTTCATGGCTGTT      900
GTTGGTATGTTGGAAGGTATGCAAATCGCTTTCTTCGCTGTTGCTAAGTT      950
GCCAGCTAACGAAAGAGGTACTTCTTTCTTCGGTAGAAAGACTTGTGAAA      1000
TCTTGTTCAAGGGTAACGGTGAAAACTTGCCAGGTTTCATGGTTGGTAGA      1050
CAATTGACTGTTGTTTGTTCTTTCTTCTTGGTTGGTTCTTTCACTTCTTT      1100
GATCATCGAACCAGGTCAAGGTGAAAACATCTTCGGTGTTTCTGACGGTG      1150
CTCAAGCTTTCTTGAACTACGGTTTCCAAGGTGCTGTTATCACTACTATC      1200
TTGGCTTCTATCACTTGGCAATTGGCTGCTTCTGCTTTCCCAATCGCTTT      1250
CTTGAACAACCCAGTTACTTTCATCTTGTTGGTTGTTGCTTTGTTCTTGG      1300
AAAGAATCGGTTTGTGTGCTGGTGCTTGGGTTTTGGCTTCTGCTCAAAAG      1350
AAGGCTATGAAGTTCGAATACGACGAAGTTTACGTTGGTACTCCAGAAGA      1400
AAGAATCGCTAACAACCACGCTGACAAGGAATACCAAGCTGGTGGTGACG      1450
TTGGTCACTTGACTGGTGGTGGTTTCACTGGTCACGTTTGTGGTTCTCAC      1500
GACGCTTTGGACGGTCCAATCGCTTCTAAGGACGCTTTGGCTGAAGACGC      1550
T

>T_pseudonana_SIT3co
ATGTGTGCTCAAAACGACGAAACTATCACTGCTTCTTCTTCTATCATCCC      50
ATCTAAGACTGCTATGATCACTAGAGACGCTGCTTTGTCTCCAACTCCAT      100
CTAACACTCCAGAAATCTACTTGTCTAAGTTGGACGACTCTGACAACAAC      150
GGTTTGGACGACCACCACGACAGAAACTTGGTTATGACTCCAATCACTGC      200
TGTTAAGTACACTTACTCTTTGGCTTTGTTGGCTTTCTCTATCATCTTGA      250
TCGTTTCTGTTATCTTCAACCAAGGTACTAAGTTGTCTACTGTTAACCCA      300
TGGTTGGCTTTGTGTGTTATGGTTGGTACTATCGTTTGGTTGGGTATGAT      350
GGAAGGTCAACAAGGTGCTTTGGTTGGTTTGACTGGTGTTGTTGACCACT      400
TGGTTTACAAGGAATCTCACTCTTTGGCTTTCAGAAACACTCAATTGGCT      450
TACAGAGGTGACAACTTGGACAGATACGTTACTGGTAGACAATTCATGGT      500
TTTGATGTGTGTTTTCGTTATCAACTTGTGTGGTTCTCCATTGCCAGGTA      550
TGTCTGAATCTTCTTTGAACTTGCCACAAATCATCGAAGAAATCTTCTTG      600
AAGACTGGTATCGCTATGATCTTGATGACTGCTATGATCTCTCAATTGCC      650
ACCACAAGTTAACGCTTCTCACTGTATGATCGACTTCATCAACAACTACT      700
TCGCTTTGTTCACTTTGTACACTGCTTTGATCATCGAATTCTCTGGTGTT      750
ATGCACGCTTCTTACTTGATCCAAAACATCATCTCTTTGATCTCTGGTAA      800
GCCAGTTAAGACTAGAGAAGAACCAAGAACTAGATTGCAAGCTTCTTTCT      850
TCTGGTTGAGAGTTTTGATGTCTTTGTCTACTTTGTGTTTCTCTATGGCT      900
GTTACTGTTGTTGCTTTGTTCCAAGGTAAGACTACTATGTGGCAAGGTGT      950
TCCAGAATGGGTTTCTTTGGTTTTGTTCTTCGTTTTGTTGACTATCGTTG      1000
GTATGTTGGAAGGTATGCAAATCGCTTTCTTGGCTACTTCTAAGATGAGA      1050
AGAGAACAAAGAGGTACTTCTTTCTTCGGTAAGAAGACTGTTGAAGTTAT      1100
CTCTAAGAAGAACGGTCAAAACTTGCCAGCTTTCTTCATCGGTAGACAAT      1150
TGATGGTTGTTGGTTGTTTCTTCATCTTGGCTAGAGTTACTACTCCAGAC      1200
GTTGAAGTTGGTACTGGTAACAACATCTTCGGTGTTTCTGACGGTGCTCA      1250
```

```
AGCTTTCTTGAACACTGGTTTGCACGCTGCTTTGTTGATGACTATCTTGG      1300
CTTCTAACACTTGGAAGTTGGCTGCTTCTACTTTCCCAGTTGCTTTCGTT      1350
AACTTGCCATTCACTTACATCTTGTTGTGGTGTGGTTTGATCTTGGAAGC      1400
TACTGGTATCTGTTCTGGTGCTTGGGTTTTGGCTAGAATCTTGAAGAGAG      1450
TTACTAAGTTGAAGTACGACGAAGAATACGTTGGTACTCCAGAAAGACCA      1500
ACTTTGTCTCAAGACTACTTGGACGCTCAAATGCCAGAATCTTTGGTTGG      1550
TCAATCTCAAGACGTTTTGGACGAAAAGCCAGGTGAAGACAGAGAAGGTG      1600
CTAAGCCACCAGAATCTAAGGTTCCATCTACTGACGCTTTGGACGAACAA      1650
GCTCAATCTCAAGGTGAATTGGACGTTGAATCTGGTTTGGCTAAG

>P_tricornutum_SIT1co
ATGGTTGACGCTGGTAACGTTATCAAGTGTGCTTACTCTGTTGGTTTGTT      50
GGTTTTCTCTACTATCATCATCATGGGTTTGATCTTCAACGAAGAAACTA      100
AGTTGTCTTCTGACGTTAACTCTGCTGTTGCTTTCGTTGCTATCTGGGGT      150
GGTGTTTTGTGGTTGACTATGGTTGAAGGTGGTCAAGGTTCTTTGGTTGG      200
TTTGGCTCCAGTTAACAGAGAATTGTACAAGGACTCTCACCCAATCGCTT      250
ACAAGTGTACTGCTATCGCTCACAAGGGTGACAACTTGGACAGATACTTG      300
TTGGGTAGACAATTCATGGTTGTTTTGACTGTTTTCGTTGTTAACATGTC      350
TGGTGGTCCATTGAAGGACGCTGAATTGTGGGGTTTCCCATACGTTTTGA      400
CTAACATGTTCTTGGGTTCTGGTTTGGCTATGATCTTGTTCACTGCTATG      450
GTTGGTCAATTGAACTCTCAAGTTAACGCTTCTTTGTGTATGTTGGACTA      500
CATCAACAACTACTTCGCTTTGTTCACTTTGTGGGTTGCTATGGCTATCG      550
AATTCTCTGGTTTGTTGCACGCTTCTTACTTGGTTCAAATGTTGGTTGCT      600
GCTTTGTCTGGTAAGAAGATCGAATCTAACGAAGAACCAAGAAACGGTTT      650
GCAAAACTTGTTCTTCTGGTCTCGTTGTTTGGGTTCTTTGGCTATCTTGG      700
GTTACTGTTTCGCTGTTACTTTGGCTGCTTTGTTCGCTGGTAAGACTACT      750
ATGTGGGAAGGTGTTCCACCATCTGTTGCTGTTATCGTTTTCTTCGTTTT      800
GATGTCTGTTGTTGGTTTGTTGGAAGGTATGCAAATCGCTTTCTTCGCTG      850
TTGCTAAGATCCCAAAGGCTGAAAGAGGTGACTCTGTTTTCGCTAAGAAG      900
ACTTGTGACTTGTTGTTCAAGGGTGACGGTAACAACTTGCCAGGTTTCAT      950
GATCGGTAGACAATTGTGTGTTGTTTCTTGTATGTTCTTCATCGCTAGAG      1000
TTACTTCTGTTGAAATCGCTGAAGGTGAAGAAAACATCTTCGGTGTTTCT      1050
GACGGTTTCCAAAAGTTGTTCGACACTGGTTTGTTGGGTGCTATCATCAC      1100
TACTATCGTTGCTTCTATCTCTTGGCAATTGGTTGCTTCTGCTTTCCCAA      1150
TCGCTTTCTTGTCTAACCCATTCACTTACATCTTCTTGAGAATCTGTTTG      1200
GTTTTGGAAGCTATCGGTATCTGTTCTGGTGCTTGGGTTTTGGCTGCTAT      1250
CCACAAGAAGGTTGCTGGTTTCCAAAGAGACGAAGTTTACATCGGTACTG      1300
CTGAAGAAAGAGCTGCTAAGGACATGTCTGACGACTCTGACCAATTGCAC      1350
TTGGGTCCAGGTCACTTGGTTAAGTTGCCAGGTTTCGCTGAACACGCTCC      1400
AACTGCTTTGAAGGACTTGATGATGGCTGACCCATCTGTTGCTGACTACT      1450
TGAAGTCTATCCACGAAATGGAATCTGGTAAGGCTAACGGTGAAGGTTCT      1500
GAAACTAGAAGAACTGGTTCTTCTGAAGCTGACGAA

>P_tricornutum_SIT2co
ATGGCTGACGTTGCTAACATCTTGAAGTGTGCTTACTCTGTTGGTTTGTT      50
GATCTTCTCTACTATCATCATCATGGGTTTGATCTTCAACGAAGAAACTA      100
AGTTGTCTTCTGACGTTCACTCTGCTGTTGCTTTCATCGCTATCTGGGTT      150
GGTGTTTTGTGGTTGACTATGGTTGAAGGTGGTCAAGGTTCTTTGGTTGG      200
TTTGGCTCCAGTTAACGGTGAATTGTACAAGGACTCTCACCCAATCGCTT      250
ACAAGTGTACTTCTATCGCTCACAAGGGTGACAACTTGGACAGATACTTG      300
TTGGGTAGACAATTCATGGTTGTTTTGACTGTTTTCACTATCAACATCTC      350
TGGTGGTCCATTGAAGGACGCTGAATTGTGGGGTTTCCCATCTGTTTTGA      400
CTAACATGTTCTTGGGTTCTGGTTTGGCTATGATCTTGTTCACTGCTATG      450
ATCGGTCAATTGAACTCTCAAGTTAACGCTTCTTTGTGTATGTTGGACTA      500
CATCAACAACTACTTCGCTTTGTTCACTTTCTGGGTTGCTATGGCTATCG      550
AATTCTCTGGTTTGTTGCACGCTTCTTACTTGGTTCAAATGTTGGTTGCT      600
GCTTTGTCTGGTAAGAAGATCGAATCTAACGAAGAACCAAGAAACGGTTT      650
GCAAAACTTGTTCTTCTGGTCTCGTTGTTTGGTTTCTTTGGCTATCTTGG      700
CTTACTGTTTCGCTGTTACTTTGGCTGCTTTGTTCGACGGTAAGACTACT      750
ATGTGGGAAGGTGTTCCATCTGCTGTTGCTGTTATCGTTTTCTTCTTGTT      800
GATGTCTGTTGTTGGTTTGTTGGAAGGTATGCAAATCGCTTTCTTCGCTG      850
TTGCTAAGATCCCAAAGTCTGAAAGAGGTGACTCTGTTTTCGCTAAGAAG      900
ACTTGTGAATTGTTGTTCAAGGGTGAAGGTAACAACTTGCCAGGTTTCAT      950
```

```
GATCGGTAGACAATTGTGTGTTGTTTCTTGTATGTTCTTCATCGCTAGAG     1000
TTACTTCTGTTGAAATCGCTGAAGGTGAAGAAAACATCTTCGGTGTTTCT     1050
GACGGTGTTCAAAAGTTGTTCGACACTGGTTTGTTGGGTGCTATCATCAC     1100
TACTATCGTTGCTTCTATCTCTTGGCAATTGGTTGCTTCTGCTTTCCCAA     1150
TCGCTTTCTTGTCTAACCCATTCACTTACATCTTCTTGAGAATCTGTTTG     1200
TTGTTGGAAGCTATCGGTATCTGTTCTGGTGCTTGGGTTTTGGCTGCTAT     1250
CCACAAGAAGATCGCTGGTTTCCAAAGAGACGAAGTTTACATCGGTACTG     1300
CTGAAGAAAGAGCTGCTAAGAACATGTCTGACAACACTGAACAATTGCAC     1350
TTGGGTGCTGGTCACTTGGTTAAGTTGCCAGGTTTCGCTGAACACGCTCC     1400
ACCAGCTTTGAAGGCTTTGATGGAAACTAACCCATCTGTTGCTGTTTACT     1450
TGAACTCTATCCACGACATGGAAACTGGTAAGGGTAACAAGGGTCAAGAA     1500
TCTGAAACTGAAACTGAA

>P_tricornutum_SIT3co
ATGTCTGACTTCTTGGACAAGTTCTGTTGTCCAGGTACTTTGGCTACTGC     50
TAACCACAACGCTAACCCAGACAGAGACGTTACTGTTGACGAACCATCTG     100
TTGACGCTACTGCTGAAGCTGACTCTCCAGAATCTACTACTACTTTCTTG     150
CCATTGGCTGACTTGCACGACTCTTCTGAACACCCAGAAACTCCAAAGGC     200
TCCAACTTCTCCACACACTGCTGCTAGAATCGGTGGTGACGAAGACTCTC     250
CAAGAATCAGAGAAGGTTTGTTGGAAAAGTTGCACGGTACTTGGATCTTG     300
CCACAAGGTGACATCCACGGTCAACCAGTTAGATACCCATCTGACGAAAT     350
CCCATACCACGCTCCACAAGACTCTAAGGAAACTGACGAATCTGACAGAA     400
AGAAGGAAACTTCTCGTTGGGGTTTGGGTTCTGGTATCGAATTGTTCAAG     450
TGTGTTTACTCTATGATCTTGTTGGTTTTCTCTATCATCGTTGTTTTGGC     500
TGCTATCTTCTCTGAACAAACTGTTGCTACTGGTGAAAACAACGTTAACC     550
CAGTTTTGGCTGTTTTCTTGGTTTGTTTCTTGATCACTTGGTTGGCTATG     600
ATGGAAGGTGGTCAAGGTGCTTTGGTTGGTTTGCAACCAATCGACAAGGA     650
ATTGTACGCTGTTTCTCACCCAAGAACTTACGCTTGTACTAAGTTGGCTC     700
ACCAAGGTAACAACATGGAAAGAGTTATCGTTGGTAGACAATTCTTGGTT     750
GTTTTGGTTGTTTTCGTTACTAACTTGATGGTTTCTTCTATCGCTAACGC     800
TTCTGTTTTGTCTTTGCCAGACGCTATCAACGAAACTTTCTTGGCTACTG     850
GTTTGGCTGTTACTTTGACTGTTATCATCGTTGGTCAATTGACTGCTCAA     900
GTTAACGCTGCTAACTGTATGTTGGACTTCATCAACAACTACTTCATGTT     950
GTTCACTATCTACACTTCTTTGGTTATCGAAGCTTCTGGTTTGTTGCACT     1000
CTGTTTACTTGGTTCAAACTATCTTCTCTAAGATGTCTGGTACTCCAATC     1050
GAATCTAACGAACCATCTCGTTCTGTTTTCCAATCTTTGTTGTTCTGGGG     1100
TAGAGTTGCTATGTCTTTGGTTTTGTTGGGTTTCTCTTTCGCTGTTTTGT     1150
TGACTGCTTTGTTCAACGGTAAGACTAACATGTGGGACGGTATCCCAGCT     1200
ATCGCTTCTGTTATCATCTTCTTCATCTTGATGGCTGTTGTTGGTATCAT     1250
GGAAGGTATGCAAATCGCTTTGTTCGCTGTTGTTAACTTGCCAAAGGAAG     1300
AATTGAGAAAGCACCCAATCGCTTACGCTAACTGTGGTTTGACTTTCTCT     1350
GGTCAAAACTTGCAAGCTTTCTTGATCGGTAGACAAATCTGTGTTACTGT     1400
TTGTACTTTCGTTATCGCTAGAATCACTTCTGTTTCTGTTAACACTGACA     1450
TCGGTGAAAACAACGTTTTCGGTGTTTCTGACGGTATCCAAAACTTCTTC     1500
AACACTGGTATGTTGGGTGCTTTGGTTACTACTATCGTTGCTTCTTTGGC     1550
TTGGAGAATCATCGCTTCTTCTTTGCCAGTTGCTTTCATGTCTAACCCAT     1600
TGATCTACTTGATCATCAGATTGTGTTTGATCTTGGAAGCTACTGGTTTG     1650
TGTTCTGCTGCTTGGGTTTTGGCTTTGTTGCAAAAGTCTTTGGCTGGTTA     1700
CCAAAGAGACGACGTTTACATCGGTACTGCTGGTGAAAGAGTTGTTTTCG     1750
CTAAGGACGGTTCTGAATTGCACTTGGAAGAACAAAACTTGAGAGCT

>C_fusiformis_SIT1co
ATGGTTTCTGTTATCGACGGTATCAAGCAATTCTACTCTATGGCTTTGGT     50
TATCTTCTCTGTTGTTATCGTTACTGCTTTGATGTTCACTGACAACACTA     100
AGTTGGCTAAGGACGCTCACCCAGTTGCTGCTTTGGTTATCATGTGGTTG     150
GGTATCTTGTGGATGTCTATGGTTGAAGGTGGTCAATGTTCTATGGTTGG     200
TTTGCCACCAATCGACAGAGACTTGTACAAGGAATCTCACCCAATCACTT     250
ACAAGATCTGTTCTTTGGGTCACAAGGGTAACAACTTGGACAGATACTTG     300
ATGGGTAGACAATTCATGGTTATCTTCATCAACTTCACTATCAACTTGTG     350
TGGTGCTCCATTGGAAGGTGCTGAAGTTTTGGGTTTGCCAGAAATCTTGA     400
CTGACATCTTCTTGGGTTCTGGTATCGCTATGGTTTTGACTGTTGTTACT     450
ATCGGTCAATTGACTGCTCAAGTTAACGCTTCTCACTGTATGTTGGACTA     500
CATCAACACTCACTTCATGACTTTCACTTTGTACGTTACTTTGGTTATCG     550
```

```
AAGCTACTGGTGTTATGCACTCTTGTTACTTGATCAGAGACATGTTCTAC      600
CACGCTGCTGGTAAGCCAGTTGAAACTAACGAACCACCAAGATCTGCTGT      650
TCAAAACTTGTTCCACTGGGGTAGAGTTGTTTTCTCTTTGGGTGTTTTGT      700
GTTTCGCTTTGGCTGTTACTATCGAAGCTTTGTTCAACGGTAAGACTACT      750
ATGTGGGAATTCATCCCAAACGGTGTTGCTATCGTTTTGTTCATCTTGTT      800
GATGTCTGTTGTTGGTTTGTTGGAAGGTATGCAAATCGCTTTCTTCGCTG      850
TTGCTAAGATCCCAAAGGCTGAAAGAGGTGACCACCCATTCGCTAGAAAG      900
ACTTGTGAATTGTTGTTCAAGGGTAAGGGTAGAAACTTGCCAGGTTTCAT      950
GGTTGGTAGACAAATGACTGTTACTTTGTGTTTCTTCATCATCGCTAGAG      1000
TTACTACTTTGGACATCGAAGTTGGTGTTGACGACAACATCTTCGGTGTT      1050
TCTGACGGTATCCAAGAATTCTTCAACTTGGGTTTCTTGGGTGCTATCAT      1100
CACTACTATCTTGGCTTCTATCGCTTGGCAATTGGTTGCTTCTGCTTTCC      1150
CAATCGCTTTCTTGTCTAACCCAATCGTTTACATCGTTTTGAGAATCGTT      1200
TTGTTGATCGAAGCTACTGGTATCTGTGCTGGTGCTTGGTTCTTGGGTAT      1250
GATCCACAAGAAGGTTGCTGGTTTCCAATTGGACGAAGTTTACGTTGGTA      1300
CTGCTGAAGAAAGAGCTGCTGGTATGAAGCCAGACCACTCTATCCACGCT      1350
GGTAGAGAATTCACTATGGGTACTAACGTTTTGAACGACAGAAAGAACTG      1400
GGAAGAAACTATCGCTAACTTGTCTGCTAAGGAAACTTTCTCTGTTAGAA      1450
GAGAAAGAATGTTGAAGAACATCAGAGAATTGAGAGCTATGGCTGAAGAA      1500
GCTTCTTCTCCAGAAGAAAAGGCTACTTTCGAAACTGCTTTGACTATGGA      1550
AACTAAGGCTTTGAACAAGTTGAACGAAGAACAAGAAAAGGAAGCTACTT      1600
TGCAAAAGGACTCTTCTGACACTGAAAACGAAGCTGACATGGCT

>C_fusiformis_SIT2co
ATGCCATCTGGTTTCCAATACTTCCAACAAGCTTACTCTATGTGTTTGGT      50
TATCTTCTCTGTTGTTATCGTTACTGCTTTGATGTTCACTGACAACACTA      100
AGTTGGCTAAGGACGCTCACCCAGTTGCTGCTTTGGTTATCATGTGGTTG      150
GGTATCTTGTGGATGTCTATGGTTGAAGGTGGTCAATGTTCTATGGTTGG      200
TTTGCCACCAATCGACAGAGACTTGTACAAGGAATCTCACCCAACTACTT      250
ACAAGATCTGTTCTTTGGGTCACAAGGGTAACAACTTGGACAGATACTTG      300
ATGGGTAGACAATTCATGGTTATCTTCATCAACTTCACTATCAACTTGTG      350
TGGTGCTCCATTGGAAGGTGCTGAAGTTTTGGGTTTGCCAGAAATCTTGA      400
CTGACATCTTCTTGGGTTCTGGTATCGCTATGGTTTTGACTGTTGTTACT      450
ATCGGTCAATTGACTGCTCAAGTTAACGCTTCTCACTGTATGTTGGACTA      500
CATCAACACTCACTTCATGACTTTCACTTTGTACGTTACTTTGATCATCG      550
AAGTTACTGGTGTTATGCACTCTTGTTACTTGATCAGAGACATGTTCTAC      600
TTCGCTGCTGGTAAGCCAGTTGAAACTAACGAACCACCAAGAAACGCTGT      650
TCAAAACTTGTTCCACTGGGGTAGAGTTGTTTTCTCTTTGGGTGTTTTGT      700
GTTTCGCTTTGGCTGTTACTTTGGAAGCTTTGTTCAAGGGTCAAACTACT      750
ATGTGGGAATTCATCCCAAACGGTGTTGCTGTTGTTTTGTTCGTTTTGTT      800
GATGTCTTTGGTTGGTTTGTTGGAAGGTATGCAAATCGCTTTCTTCGCTG      850
TTGCTAAGATCCCAAAGGCTGACAGAGGTGACCACCCATTCGCTAGAAAG      900
ACTTGTGAAGTTTTGTTCAAGGGTAACGGTAGAAACTTGCCAGGTTTCAT      950
GGTTGGTAGACAAATGACTGTTACTTTGTGTTTCTTCATCATCGCTAGAG      1000
TTACTACTTTGGACATCGAAATCGGTGTTGACGACAACATCTTCGGTGTT      1050
TCTGACGGTATCCAAGAATTCTTCAACTTGGGTTTCTTGGGTGCTATCAT      1100
CACTACTATCTTGGCTTCTATCGCTTGGCAATTGGTTGCTTCTGCTTTCC      1150
CAATCGCTTTCTTGTCTAACCCAGTTGTTTACATCGTTTTGAGAATCGTT      1200
TTGTTGATCGAAGCTACTGGTATCTGTGCTGGTGCTTGGTTCTTGGGTAT      1250
GATCCACAAGAAGGTTGCTGGTTTCCAATTGGACGAAGTTTACGTTGGTA      1300
CTGCTGAAGAAAGAGCTGCTGGTCAAAAGCCAGACCACTCTGTTCACGCT      1350
GGTAGAGACTTCACTATCGGTACTAACGTTTTGTCTAAGCCACCAGCTAA      1400
CTGGGAAGAAGCTTTGGCTAACTTGGGTCCAGTTCCAGAAACTTTCTCTC      1450
AAAGAAGAGAAAGAATCTTGAAGAACATCAAGGAAATGAAGGAATTGATG      1500
GGTAACGCTGGTACTGACGCTGAACAATCTGCTTACGAAGCTGGTTTGGC      1550
TGCTGAAGTTAAGGCTTTGCAAAAGTTGAACCAAGAAGAATCTGAAGCTG      1600
CTGACCCAACTAAGGGTGTTGACTCTGTT

>C_fusiformis_SIT3co
ATGGTTTCTGTTATCGACGGTATCAAGCAATTCTACTCTATGGCTTTGGT      50
TATCTTCTCTGTTGTTATCGTTACTGCTTTGATGTTCACTGACAACACTA      100
AGATGGCTAAGGACGCTCACCCAGTTGCTGCTTTGGTTATCATGTGGTTG      150
GGTATCTTGTGGATGTCTATGGTTGAAGGTGGTCAATGTTCTATGGTTGG      200
```

TTTGCCACCAATCGACAGAGAATTGTACAAGGAATCTCACCCAATCACTT 250
ACAAGATCTGTTCTTTGGGTCACAAGGGTAACAACTTGGACAGATACTTG 300
ATGGGTAGACAATTCTTGGTTATCTTCATCAACTTCACTATCAACTTGTG 350
TGGTGCTCCATTGGACGGTGCTGAAGTTTTGGGTTTGCCAAAGGTTTTGA 400
CTGACATCTTCTTGGGTTCTGGTATCGCTATGGTTTTGATCGTTGTTCCA 450
TTGGGTCAATTGACTGCTCAAGTTAACTCTTCTCACTGTATGTTGGACTA 500
CATCAACACTCACTTCATGACTTTCACTTTGTACATCACTTTGATCATCG 550
AAGCTACTGGTGTTATGCACGTTTGTTACTTGATCAGAGACATGTTCTAC 600
CACGCTGCTGGTAAGCCAGTTGAATCTAACGAACCACCAAGATCTGCTGT 650
TCAAAACGTTTTCCACTGGGGTAGAGTTGCTTTCTCTTTGGGTGTTTTGT 700
GTTTCGCTTTGGCTGTTACTATCGAAGCTTTGTTCAACGGTGAAACTACT 750
ATGTGGCAATCTATCCCAAACGGTGTTGCTATCGTTTTGTTCATCTTGTT 800
GATGGCTGTTGTTGGTTTGTTGGAAGGTATGCAAATCGCTTTCTTCGCTG 850
TTGCTAAGATCCCAAAGGCTGAAAGAGGTGACCACCCATTCGCTAGAAAG 900
ACTTGTGAATTGGTTTTCAGAGGTAACGGTAGAAACTTGCCAGGTTTCAT 950
GGTTGGTAGACAAATCACTGTTACTTTGTGTTTCTTCATCATCGCTAGAG 1000
TTACTACTTTGGACGTTGACGTTGGTGTTGACGACAACATCTTCGGTGTT 1050
TCTGACGGTGTTCAAGAATTCTTCAACTTGGGTTTCTTGGGTGCTATCAT 1100
CACTACTATCTTGGCTTCTATCGCTTGGCAATTGGTTGCTTCTGCTTTCC 1150
CAATCGCTTTCTTGTCTAACCCAATCGTTTACATCATGTTGAGAATCGTT 1200
TTGTTGATCCAATCTACTGGTATCTGTGCTTCTGCTTGGTTCTTGGGTAT 1250
GATCCACAAGAAGATCGCTGGTTTCCAAGAAGACGAAGTTTACGTTGGTA 1300
CTGCTGAAGAAAGAGCTGCTGGTGAAAAGCCAGACCACTCTGTTCACCCA 1350
GGTAGAGAATTCACTATGGGTACTAACGTTTTGAACGACGGTAGAAACTG 1400
GGACGAAGCTATCGCTAACTTGTCTGCTTTGGAAACTTTCTCTGTTAAGT 1450
TGGAAAGAATGTTGAAGAACATCAGAGAATTGAGAGAAATGATCAACTCT 1500
TCTGACATCTCTGCTGAAGAAAGAACTACTTTCGAAAAGGCTTTGGCTAT 1550
CGAAGTTAGAGCTTTGGACAAGTTGAACGCTGAAGAAGAAGAAGCTACTA 1600
ACAAGTCTGAAGGTAAGGACACTGAAGCTTCTAAGGAAACTGACGCTTCT 1650
GACGCTGAATTGGGTGAAGCT

>T_oceanica_SITco
ATGTCTGCTGCTGAAGTTGAAAAGTCTTCTTCTCACGCTGACGCTCACGA 50
CGTTAAGTTGACTCCATTGTTGGCTTTCAAGTACGTTGCTTCTTTGGGTT 100
TGATGGTTTTCTCTATGGTTTTGGTTGGTGCTTTGATCTTCACTAAGAAC 150
ACTAGAGTTGCTCAAGAAGCTTCTCCATGGGTTTCTTTGATCGTTTTGAT 200
CTTGGCTGTTTTGTGGTTGACTATGATCGAAGGTCAACAAGCTTCTTTGG 250
TTGGTTTGCCACCAGTTGACCCAGACTTGTACAAGGACTCTCACCCAATG 300
ACTTACAAGAACGCTGCTATCGCTTTCAAGGGTGACAACTTGGACAGATA 350
CTTGATGGGTAGACAATTCATGGTTTTGTTGGTTGTTTTCGTTATCAACC 400
AATGTGCTTCTCCAATGGACCCATCTGCTGACGTTTTGGGTTTGCCAGAC 450
GGTGTTAAGACTGTTTTCTTGGACGTTCCAGGTTTGGGTATGATCATCTT 500
CACTTGTATCTTGGGTCAATTGACTACTCAAGTTAACGCTTCTCACTGTA 550
TGATCGACACTATCAACAACTACTTCGCTTTGTTCACTTTGTACACTACT 600
ATGGTTGTTGAATTCTCTGGTATCATGCACGCTTCTTACTTGATCCAAAA 650
CATCTTGTCTGGTATCTCTGGTAAGCCAATCATGACTAACGAAGAACCAA 700
GAAAGGGTTTCACTTTCGCTTTCTTCTGGGGTAGAGTTGTTATGTCTGTT 750
GCTATCTTGTCTTTCTGTATGGCTGCTACTTTGGTTGCTTTGTTCAACGG 800
TCAAACTTCTATCTCTACTAAGTACCCATCTATCACTCCAGGTATCTCTG 850
TTTTCTTGTTCTTCTTCTTCATGGCTATCGTTGGTATGTTGGAAGGTATG 900
CAAATCGCTTTCTTCGCTGTTGCTAAGTTGCCAGCTAACGAAAGAGGTAC 950
TTCTTTCTTCGGTAGAAAGACTTGTGAATTGTTGTTCAAGGGTAACGGTG 1000
AAAACTTGCCAGGTTTCATGATCGGTAGACAATTGACTGTTGTTGCTTCT 1050
TTCTTCTTGGTTGGTTCTATCACTTCTATGAACATCGTTCCAGGTACTGG 1100
TGAAAACATCTTCGGTGTTTCTGACTCTGCTCAAACTTTCTTGAACTACG 1150
GTTTCCAAGGTGCTGTTATCACTACTATCTTGGCTTCTATCTCTTGGCAA 1200
TTGGCTGCTTCTGCTTTCCCAATCGCTTTCTTGAACAACCCAGTTACTTA 1250
CATCTTGTTGTGTGTTGCTTTGTTCTTGGAATTCACTGGTTTGTGTTCTG 1300
GTGCTTGGGTTTTGGCTAGAGTTTTGAAGACTACTTTGAAGTACCAATAC 1350
GACGAAGTTTACGTTGGTACTCCAGAAGAAAGAGCTCAAAACAACCACGC 1400
TGACAAGGCTTTCGGTGACGACATGGGTCACTTGGCTGGTGCTGGTTTCA 1450
GAGGTTACGCTTGTGGTTCTCGTGACGCTTTGGACGGTCCAATCGAATTG 1500
CCAGAAGCTGTTAACGACGACTCTTTCTACCCAGGTATGGAAGGTCCAGT 1550

```
TTTGTCTAAGGACGCTTTGGACCCA

>S_costatum_SITco
ATGACTGACGCTGAAATCAAGAAGGAAGAATTGGCTGACGCTCACGACGT      50
TGAATTGACTCCATTCACTATCTTCAGATACACTTACTCTGTTATCTTGT      100
TGATCTTCTCTATCGTTTTGGTTGTTTCTTTGATGTTCACTGGTAACACT      150
AAGTTGGCTGCTGACGCTTCTCCATGGGCTGCTTTGTTCGTTTGTATCGC      200
TGCTGTTGTTTGGTTGTCTATGATCGAAGGTCAACAAGCTTCTTTGGTTG      250
GTTTGCCACCAGTTGACCCAGAATTGTACAAGGAAACTCACCCAGTTACT      300
TACTTGAACGCTGCTACTGCTTTCTTGGGTGACAACTTGGACAGATACTT      350
GATGGGTAGACAATTCATGGTTTTGTTGGTTGTTTTCATCATCAACTTGT      400
GTGGTGCTCCATCTTCTGGTGACGCTGACGTTTTGGGTATGCCAGGTTGG      450
TTGAAGACTATCTTCTTGGACGTTGGTTTGGGTATGATCATCTTCACTTG      500
TCAATTGGGTCAATTGACTACTCAAGTTAACGCTTCTCACTGTATGTTGG      550
ACTTCATCAACAACTACTTCGCTTTGTTCACTTTGTACACTGCTATGTGT      600
ATCGAATTCTCTGGTGTTATGCACTCTTCTTACTTGATCCAAAACGTTTT      650
GTCTTTCGCTTCTGGTAAGCCAATCCACTCTAACGAAGAACCAAAGAGAG      700
GTTTCACTTTGTTGTTCTTCTGGGGTAGAGTTTTGATGTCTTTGGCTATC      750
TTGGGTTTCTCTTTGGCTGTTGTTATCTCTGCTTTGTTCCAAGGTAGAAC      800
TATGATGGCTGTTAAGTACCCATCTGTTTCTAACGGTGCTTCTGTTTTCT      850
TGTTCTTCTTCTTGATGTGTATCGTTGGTATGTTGGAAGGTATGCAAATC      900
GCTTTCTTCGCTGTTGCTAAGTTGCCAGCTTCTGAAAGAGGTACTACTTT      950
CTTCGGTAGAAAGACTTGTGACTTGTTGTTCAAGGGTAACGGTCAAAACT      1000
TGCCAGGTTTCATGATCGGTAGACAATTGACTGTTGTTGCTTCTTTCTTC      1050
ATCGTTGCTTCTATCACTTCTATGAACATCCAACCAGGTAACGAAGACGG      1100
TAACATCTTCGGTGTTTCTGACGGTGCTCAAGCTTTCTTGAACTTGGGTT      1150
TCCACGCTGCTGTTATCACTACTATCTTGGCTTCTATCACTTGGCAATTG      1200
GCTGCTTCTGCTTTCCCAATCGCTTTCTTGAACAACCCAGTTACTTACGT      1250
TTTGTTGGTTTTCGCTTTGTTCTTGGAATGGACTGGTTTGTGTGCTGGTG      1300
CTTGGGTTTTGGCTAGAGTTATGAAGAAGGCTTTGAAGTACGAATACGAC      1350
GAAGTTTACGTTGGTACTCCAGAAGAAAGAGCTGCTAACAACCACGCTGA      1400
CAAGGACTTCGCTGACGACACTGGTAAGATGTACGGTGGTGGTTTCAGAG      1450
GTCACGCTGTTGGTTCTCACGACGCTTTGGACGGTCCAATCGCTTCTAAG      1500
GACGAAGTTGAAGAAGAAGCTGTT

>N_alba_SITco
ATGCAACACTCTACTTGGAACTACATCCAAATGGCTTACTCTATGGGTTT      50
GGTTATCTTCTCTGTTATCATCGTTACTGCTTTGATGTTCGAAAAGGACA      100
CTAAGATCGCTAACGACGTTCACCCAGCTGCTGCTTTGGTTATCATGTGG      150
GCTGGTATCTTGTGGATGTCTATGGTTGAAGGTGGTCAATGTTCTATGGT      200
TGGTTTGCCACCAGTTAACAGAGACTTGTACAAGGAATCTCACCCAATCA      250
CTCACCAAATCTGTTCTTTGGGTCACAAGGGTGACAACTTGGACAGATAC      300
TTGATGGGTAGACAATTCATGGTTATCTTCATCAACTTCACTATCAACTT      350
GTGTGGTGCTCCATTGGAAGACGCTGAAGTTTTGGGTTTGCCATCTGTTA      400
TCCAATCTATCTTCTTGGGTTCTGGTATCGCTATGATCTTGACTGTTGTT      450
ACTATCGGTCAATTGACTGCTCAAGTTAACGCTTCTCACTGTATGTTGGA      500
CTACGTTAACACTCACTTCATGACTTTCACTTTGTACGTTGCTTTGGCTA      550
TCGAAAAGACTGGTGTTAAGCACACTTCTTACTTGATCCAATACTTCTTC      600
TACTGGTTGGCTGGTAAGCCAGTTGTTACTAACGAACCACCAAGATCTGC      650
TCCACAAGCTATCTTCTTCTGGGGTAGATGTTTGTTCTCTGTTGGTGTTT      700
TGTTCTTCGCTTTGGCTGTTACTTTGAAGGCTTTGTTCGACGGTAACACT      750
ACTATGTGGTCTTTCATCCCAAACACTGTTGCTGTTATCTTGTTCTTCTT      800
GTTGATGTCTGTTGTTGGTTTGTTGGAAGGTATGCAAATCGCTTTCTTCG      850
CTGTTGCTAAGTTGAGAAAGGAAGAAAGAGGTGAACACCCAATGGCTATG      900
AGAACTTGTGAATTGTTGTTCAGAGGTGAAGGTAAGAACTTGCCAGGTTT      950
CATGGTTGGTAGACAAATGACTGTTACTTTGTGTTTCTTCGTTATCGCTA      1000
GAGTTACTACTTTGGACATCGAAGTTGGTGTTGACGAAAACGTTTTCGGT      1050
GTTTCTGACCCAATCCAAGAATTCTTCAACATGGGTTTCTTGGGTGCTAT      1100
CATCACTGCTATCTTGGGTTCTATCGCTTGGCAATTGGTTGCTTCTGCTT      1150
TCCCATTGGAATTCTTGTCTAACCCAATGGTTTACATCTTCTTGAACTTG      1200
GCTTTGGCTTTGGAAGCTACTGGTGTTGTTTCTGGTGCTTGGTTCTTGGG      1250
TATCATCCACAAGAACGTTGCTGGTTTCCAAGAAGACGAAGTTTACATCG      1300
GTACTCCAGAAGAAAGAGCTGCTATGGACAAGGCTGACTTGGCTTCTGAA      1350
```

```
AAGGGTGACTCTGTTGCTGAAGCTCACATGGGTACTAACATGTTGAACTT      1400
GCCACCAGGTTCTAAGGGTATCCCAGCTGAATGGAGATCTCAAAAGTTCA      1450
ACTACTCTAAGTCTTACTCTGAACAAAGAGCTGACATCTTGTCTAACATC      1500
AAGGACTTGAGAGAACAAATCGGTATGGCTGGTTCTAAGGAAGAAAGAGA      1550
AGCTTTCGAAGGTGCTTTGGCTATGGAAACTCAAGCTTTGTTGAAGGTTA      1600
ACAAGGAACAACAAGAAGCTGAAGACTTGGTTTTGGTTGAAGAAGAA
```

Supplementary Figure 1. Yeast codon-optimized cDNA sequences of the SIT homologues expressed in this study.

2. MULTIPLE SEQUENCE ALIGNMENT

```
CLUSTAL O(1.2.1) multiple sequence alignment


TpSIT1          ------------------------------------------------------------ 0
TpSIT3          -------MCA--------------------------------------------QND--- 6
CfSIT1          ------------------------------------------------------------ 0
CfSIT2          ------------------------------------------------------------ 0
CfSIT3          ------------------------------------------------------------ 0
PtSIT1          ------------------------------------------------------------ 0
PtSIT2          ------------------------------------------------------------ 0
PtSIT3          MSDFLDKFCCPGTLATANHNANPDRDVTVDEPSVDATAEADSPESTTTFLPLADLHDSSE 60
ToSIT           ------------------------------------------------------------ 0
ScSIT           ------------------------------------------------------------ 0
NaSIT           ------------------------------------------------------------ 0


TpSIT1          ---------------------------------------------------MSTAEI-QS 8
TpSIT3          ETITASSSIIPSKTAM----------------------ITRDAALS--PTPSNTPEIYLS 42
CfSIT1          ------------------------------------------------------------ 0
CfSIT2          ------------------------------------------------------------ 0
CfSIT3          ------------------------------------------------------------ 0
PtSIT1          ------------------------------------------------------------ 0
PtSIT2          ------------------------------------------------------------ 0
PtSIT3          HPETPKAPTSPHTAARIGGDEDSPRIREGLLEKLHGTWILPQGDIHGQPVRYPSDEIPYH 120
ToSIT           ---------------------------------------------------MSA------ 3
ScSIT           ------------------------------------------------------------ 0
NaSIT           ------------------------------------------------------------ 0


TpSIT1          GADTAP---VKHDDHADSHDVKLTPFNILRYIGSIGLLIFSIIIVGALMFTGNTRVA--K 63
TpSIT3          KLDDSD---NNGLDDHHDRNLVMTPITAVKYTYSLALLAFSIILIVSVIFNQGTKLS--- 96
CfSIT1          ---------------------MVSVIDGIKQFYSMALVIFSVVIVTALMFTDNTKLA--K 37
CfSIT2          ---------------------MPSGFQYFQQAYSMCLVIFSVVIVTALMFTDNTKLA--K 37
CfSIT3          ---------------------MVSVIDGIKQFYSMALVIFSVVIVTALMFTDNTKMA--K 37
PtSIT1          ---------------------MVDAGNVIKCAYSVGLLVFSTIIIMGLIFNEETKLS--S 37
PtSIT2          ---------------------MADVANILKCAYSVGLLIFSTIIIMGLIFNEETKLS--S 37
PtSIT3          APQDSKETDESDRKKETSRWGLGSGIELFKCVYSMILLVFSIIVVLAAIFSEQTVATGEN 180
ToSIT           ---AEV---EKSSSHADAHDVKLTPLLAFKYVASLGLMVFSMVLVGALIFTKNTRVA--Q 55
ScSIT           -MTDAE---IKKEELADAHDVELTPFTIFRYTYSVILLIFSIVLVVSLMFTGNTKLA--A 54
NaSIT           --------------------MQHSTWNYIQMAYSMGLVIFSVIIVTALMFEKDTKIA--N 38
                                            .:   *: *: ** ::: . :*   *  :

TpSIT1          DANPWVSLIVCILAIVWLSMIEGQQASLVGLPPV-DPDLYKDSHPLTYKNAALAFKGDNL 122
TpSIT3          TVNPWLALCVMVGTIVWLGMMEGQQGALVGLTGVVDHLVYKESHSLAFRNTQLAYRGDNL 156
CfSIT1          DAHPVAALVIMWLGILWMSMVEGGQCSMVGLPPI-DRDLYKESHPITYKICSLGHKGNNL 96
CfSIT2          DAHPVAALVIMWLGILWMSMVEGGQCSMVGLPPI-DRDLYKESHPTTYKICSLGHKGNNL 96
CfSIT3          DAHPVAALVIMWLGILWMSMVEGGQCSMVGLPPI-DRELYKESHPITYKICSLGHKGNNL 96
PtSIT1          DVNSAVAFVAIWGGVLWLTMVEGGQGSLVGLAPV-NRELYKDSHPIAYKCTAIAHKGDNL 96
PtSIT2          DVHSAVAFIAIWVGVLWLTMVEGGQGSLVGLAPV-NGELYKDSHPIAYKCTSIAHKGDNL 96
PtSIT3          NVNPVLAVFLVCFLITWLAMMEGGQGALVGLQPI-DKELYAVSHPRTYACTKLAHQGNNM 239
ToSIT           EASPWVSLIVLILAVLWLTMIEGQQASLVGLPPV-DPDLYKDSHPMTYKNAAIAFKGDNL 114
ScSIT           DASPWAALFVCIAAVVWLSMIEGQQASLVGLPPV-DPELYKETHPVTYLNAATAFLGDNL 113
NaSIT           DVHPAAALVIMWAGILWMSMVEGGQCSMVGLPPV-NRDLYKESHPITHQICSLGHKGDNL 97
                 .    :.      : *: *:** * ::***  : :  :*  :*  :.     .. *:*:

TpSIT1          DRYLMGRQFMVLLVVFVINQCSSPLDPTV-DVLGLPDGVKFIFLDI-GLAMIIFTCILGQ 180
TpSIT3          DRYVTGRQFMVLMCVFVINLCGSPLPGMSESSLNLPQIIEEIFLKT-GIAMILMTAMISQ 215
CfSIT1          DRYLMGRQFMVIFINFTINLCGAPLEG-A-EVLGLPEILTDIFLGS-GIAMVLTVVTIGQ 153
CfSIT2          DRYLMGRQFMVIFINFTINLCGAPLEG-A-EVLGLPEILTDIFLGS-GIAMVLTVVTIGQ 153
CfSIT3          DRYLMGRQFLVIFINFTINLCGAPLDG-A-EVLGLPKVLTDIFLGS-GIAMVLIVVPLGQ 153
PtSIT1          DRYLLGRQFMVVLTVFVVNMSGGPLKD-A-ELWGFPYVLTNMFLGS-GLAMILFTAMVGQ 153
PtSIT2          DRYLLGRQFMVVLTVFTINISGGPLKD-A-ELWGFPSVLTNMFLGS-GLAMILFTAMIGQ 153
PtSIT3          ERVIVGRQFLVVLVVFVTNLMVSSIAN-A-SVLSLPDAINETFLAT-GLAVTLTVIIVGQ 296
ToSIT           DRYLMGRQFMVLLVVFVINQCASPMDPSA-DVLGLPDGVKTVFLDVPGLGMIIFTCILGQ 173
ScSIT           DRYLMGRQFMVLLVVFIINLCGAPSSGDA-DVLGMPGWLKTIFLDV-GLGMIIFTCQLGQ 171
NaSIT           DRYLMGRQFMVIFINFTINLCGAPLED-A-EVLGLPSVIQSIFLGS-GIAMILTVVTIGQ 154
                :* : ****:*::  *  *   .       .   :*  :   **   *:.: : .  :.*
```

```
TpSIT1      LTTQVNASYAMIDFINNYFALFTLYTTMAVEFSGIMHSSYLIQNILSAVSGKPIQTNEEP  240
TpSIT3      LPPQVNASHCMIDFINNYFALFTLYTALIIEFSGVMHASYLIQNIISLISGKPVKTREEP  275
CfSIT1      LTAQVNASHCMLDYINTHFMTFTLYVTLVIEATGVMHSCYLIRDMFYHAAGKPVETNEPP  213
CfSIT2      LTAQVNASHCMLDYINTHFMTFTLYVTLIIEVTGVMHSCYLIRDMFYFAAGKPVETNEPP  213
CfSIT3      LTAQVNSSHCMLDYINTHFMTFTLYITLIIEATGVMHVCYLIRDMFYHAAGKPVESNEPP  213
PtSIT1      LNSQVNASLCMLDYINNYFALFTLWVAMAIEFSGLLHASYLVQMLVAALSGKKIESNEEP  213
PtSIT2      LNSQVNASLCMLDYINNYFALFTFWVAMAIEFSGLLHASYLVQMLVAALSGKKIESNEEP  213
PtSIT3      LTAQVNAANCMLDFINNYFMLFTIYTSLVIEASGLLHSVYLVQTIFSKMSGTPIESNEPS  356
ToSIT       LTTQVNASHCMIDTINNYFALFTLYTTMVVEFSGIMHASYLIQNILSGISGKPIMTNEEP  233
ScSIT       LTTQVNASHCMLDFINNYFALFTLYTAMCIEFSGVMHSSYLIQNVLSFASGKPIHSNEEP  231
NaSIT       LTAQVNASHCMLDYVNTHFMTFTLYVALAIEKTGVKHTSYLIQYFFYWLAGKPVVTNEPP  214
            *  ***:: .*:* :*.:*  **:: :: :* :*: *  **:: ..   :*. : :.*

TpSIT1      KTGMTFAFFWGRVLMSLAILGFCLAVTLVALFNGQTSVSVKYPSISPGLSVFLLFFFMAV  300
TpSIT3      RTRLQASFFWLRVLMSLSTLCFSMAVTVVALFQGKTTM---WQGVPEWVSLVLFFVLLTI  332
CfSIT1      RSAVQNLFHWGRVVFSLGVLCFALAVTIEALFNGKTTM---WEFIPNGVAIVLFILLMSV  270
CfSIT2      RNAVQNLFHWGRVVFSLGVLCFALAVTLEALFKGQTTM---WEFIPNGVAVVLFVLLMSL  270
CfSIT3      RSAVQNVFHWGRVAFSLGVLCFALAVTIEALFNGETTM---WQSIPNGVAIVLFILLMAV  270
PtSIT1      RNGLQNLFFWSRCLGSLAILGYCFAVTLAALFAGKTTM---WEGVPPSVAVIVFFVLMSV  270
PtSIT2      RNGLQNLFFWSRCLVSLAILAYCFAVTLAALFDGKTTM---WEGVPSAVAVIVFFLLMSV  270
PtSIT3      RSVFQSLLFWGRVAMSLVLLGFSFAVLLTALFNGKTNM---WDGIPAIASVIIFFILMAV  413
ToSIT       RKGFTFAFFWGRVVMSVAILSFCMAATLVALFNGQTSISTKYPSITPGISVFLFFFFMAI  293
ScSIT       KRGFTLLFFWGRVLMSLAILGFSLAVVISALFQGRTMMAVKYPSVSNGASVFLFFFLMCI  291
NaSIT       RSAPQAIFFWGRCLFSVGVLFFALAVTLKALFDGNTTM---WSFIPNTVAVILFFLLMSV  271
            :      :.* *   *:  * :.:*. : *** *.* :   :  :    ::.::..:: :

TpSIT1      VGMLEGMQIAFFAVAKLPANERGTSFFGRKTCEILFKGNGENLPGFMVGRQLTVVCSFFL  360
TpSIT3      VGMLEGMQIAFLATSKMRREQRGTSFFGKKTVEVISKKNGQNLPAFFIGRQLMVVGCFFI  392
CfSIT1      VGLLEGMQIAFFAVAKIPKAERGDHPFARKTCELLFKGKGRNLPGFMVGRQMTVTLCFFI  330
CfSIT2      VGLLEGMQIAFFAVAKIPKADRGDHPFARKTCEVLFKGNGRNLPGFMVGRQMTVTLCFFI  330
CfSIT3      VGLLEGMQIAFFAVAKIPKAERGDHPFARKTCELVFRGNGRNLPGFMVGRQITVTLCFFI  330
PtSIT1      VGLLEGMQIAFFAVAKIPKAERGDSVFAKKTCDLLFKGDGNNLPGFMIGRQLCVVSCMFF  330
PtSIT2      VGLLEGMQIAFFAVAKIPKSERGDSVFAKKTCELLFKGEGNNLPGFMIGRQLCVVSCMFF  330
PtSIT3      VGIMEGMQIALFAVVNLPKEELRKHPIAYANCGLTFS--GQNLQAFLIGRQICVTVCTFV  471
ToSIT       VGMLEGMQIAFFAVAKLPANERGTSFFGRKTCELLFKGNGENLPGFMIGRQLTVVASFFL  353
ScSIT       VGMLEGMQIAFFAVAKLPASERGTTFFGRKTCDLLFKGNGQNLPGFMIGRQLTVVASFFI  351
NaSIT       VGLLEGMQIAFFAVAKLRKEERGEHPMAMRTCELLFRGEGKNLPGFMVGRQMTVTLCFFV  331
            **::******::*. ::   :     :.  .  :     *.** .*::***: *. . *.

TpSIT1      VGSFTSLIIEPGQG-ENIFGVSDGAQAFLNYGFQGAVITTILASITWQLAASAFPIAFLN  419
TpSIT3      LARVTTPDVEVGTG-NNIFGVSDGAQAFLNTGLHAALLMTILASNTWKLAASTFPVAFVN  451
CfSIT1      IARVTTLDIEVGVD-DNIFGVSDGIQEFFNLGFLGAIITTILASIAWQLVASAFPIAFLS  389
CfSIT2      IARVTTLDIEIGVD-DNIFGVSDGIQEFFNLGFLGAIITTILASIAWQLVASAFPIAFLS  389
CfSIT3      IARVTTLDVDVGVD-DNIFGVSDGVQEFFNLGFLGAIITTILASIAWQLVASAFPIAFLS  389
PtSIT1      IARVTSVEIAEG-E-ENIFGVSDGFQKLFDTGLLGAIITTIVASISWQLVASAFPIAFLS  388
PtSIT2      IARVTSVEIAEG-E-ENIFGVSDGVQKLFDTGLLGAIITTIVASISWQLVASAFPIAFLS  388
PtSIT3      IARITSVSVNTDIGENNVFGVSDGIQNFFNTGMLGALVTTIVASLAWRIIASSLPVAFMS  531
ToSIT       VGSITSMNIVPGTG-ENIFGVSDSAQTFLNYGFQGAVITTILASISWQLAASAFPIAFLN  412
ScSIT       VASITSMNIQPGNEDGNIFGVSDGAQAFLNLGFHAAVITTILASITWQLAASAFPIAFLN  411
NaSIT       IARVTTLDIEVGVD-ENVFGVSDPIQEFFNMGFLGAIITAILGSIAWQLVASAFPLEFLS  390
            :. .*:  :        *:*****  * ::: *: .*:: :*:.* :*:: **::*: *:.

TpSIT1      NPVTFILLVVALFLERIGLCAGAWVLASAQKKAMKFEYDEVYVGTPEERIANNHADKEYQ  479
TpSIT3      LPFTYILLWCGLILEATGICSGAWVLARILKRVTKLKYDEEYVGTPERPTLSQDYLDAQM  511
CfSIT1      NPIVYIVLRIVLLIEATGICAGAWFLGMIHKKVAGFQLDEVYVGTAEERAAGMKPDHSIH  449
CfSIT2      NPVVYIVLRIVLLIEATGICAGAWFLGMIHKKVAGFQLDEVYVGTAEERAAGQKPDHSVH  449
CfSIT3      NPIVYIMLRIVLLIQSTGICASAWFLGMIHKKIAGFQEDEVYVGTAEERAAGEKPDHSVH  449
PtSIT1      NPFTYIFLRICLVLEAIGICSGAWVLAAIHKKVAGFQRDEVYIGTAEERAAKDMSDDSDQ  448
PtSIT2      NPFTYIFLRICLLLEAIGICSGAWVLAAIHKKIAGFQRDEVYIGTAEERAAKNMSDNTEQ  448
PtSIT3      NPLIYLIIRLCLILEATGLCSAAWVLALLQKSLAGYQRDDVYIGTAGERVVFAKDGSELH  591
ToSIT       NPVTYILLCVALFLEFTGLCSGAWVLARVLKTTLKYQYDEVYVGTPEERAQNNHADKAF-  471
ScSIT       NPVTYVLLVFALFLEWTGLCAGAWVLARVMKKALKYEYDEVYVGTPEERAANNHADKDF-  470
NaSIT       NPMVYIFLNLALALEATGVVSGAWFLGIIHKNVAGFQEDEVYIGTPEERAAMDKADLASE  450
             *. ::.:    * ::  *: :.**.*.    *      : *: *:**  .
```

```
TpSIT1      AGGDVGHLTGGG-F----TG----------HVCGSHDALDGPIASKDALAEDA-------  517
TpSIT3      PESLVGQ---SQDVLDEKPGEDREGAKPPESKVPSTDALDEQAQSQGELD----------  558
CfSIT1      AGR---EFTMGTNVLND-RK-----------------NWEETIANLSA-K-ETFSVRRER  486
CfSIT2      AGR---DFTIGTNVLSKPPA-----------------NWEEALANLGPVP-ETFSQRRER  488
CfSIT3      PGR---EFTMGTNVLND-GR-----------------NWDEAIANLSA-L-ETFSVKLER  486
PtSIT1      -------LHLGPGHLVKLPG----------FAEHAPTALKDLMM---------ADPSVAD  482
PtSIT2      -------LHLGAGHLVKLPG----------FAEHAPPALKALME---------TNPSVAV  482
PtSIT3      LEEQ---------NLRA-------------------------------------------  599
ToSIT       -GDDMGHLAGAG-F----RG----------YACGSRDALDGPIELPEAVNDDSFYPGMEG  515
ScSIT       -ADDTGKMYGGG-F----RG----------HAVGSHDALDGPIASKDEVEEEAV------  508
NaSIT       KGDSVAEAHMGTNMLNLPPG-----------SKGIPAEWRSQKFNY---S-KSYSEQRAD  495


TpSIT1      ------------------------------------------------------------  517
TpSIT3      ------------------------VESGLAK-----------------------------  565
CfSIT1      MLKNIRELRAMAEEA-SSPEEKATFETALTMETKALNKLNEEQEKEATLQKDSSDTENEA  545
CfSIT2      ILKNIKEMKELMGNAGTD-AEQSAYEAGLAAEVKALQKLNQEESEAADPTKGVDSV----  543
CfSIT3      MLKNIRELREMINSSDISAEERTTFEKALAIEVRALDKLNAEEEEATNKS-EGKDTEASK  545
PtSIT1      YLKSIHEMESGKANGEGSETRRTGSSE--ADE----------------------------  512
PtSIT2      YLNSIHDMETGKGNKGQESETETE------------------------------------  506
PtSIT3      ------------------------------------------------------------  599
ToSIT       PVLSKDALDP--------------------------------------------------  525
ScSIT       ------------------------------------------------------------  508
NaSIT       ILSNIKDLREQIGMAGSK-EEREAFEGALAMETQALLKVNKEQQEAEDLVLVEEE-----  549


TpSIT1      ------------  517
TpSIT3      ------------  565
CfSIT1      --DMA-------  548
CfSIT2      ------------  543
CfSIT3      ETDASDAELGEA  557
PtSIT1      ------------  512
PtSIT2      ------------  506
PtSIT3      ------------  599
ToSIT       ------------  525
ScSIT       ------------  508
NaSIT       ------------  549
```

Supplementary Figure 2. Multiple sequence alignment of SIT homologues. See also Supplementary Note 1.

3. TOPOLOGY PREDICTION.

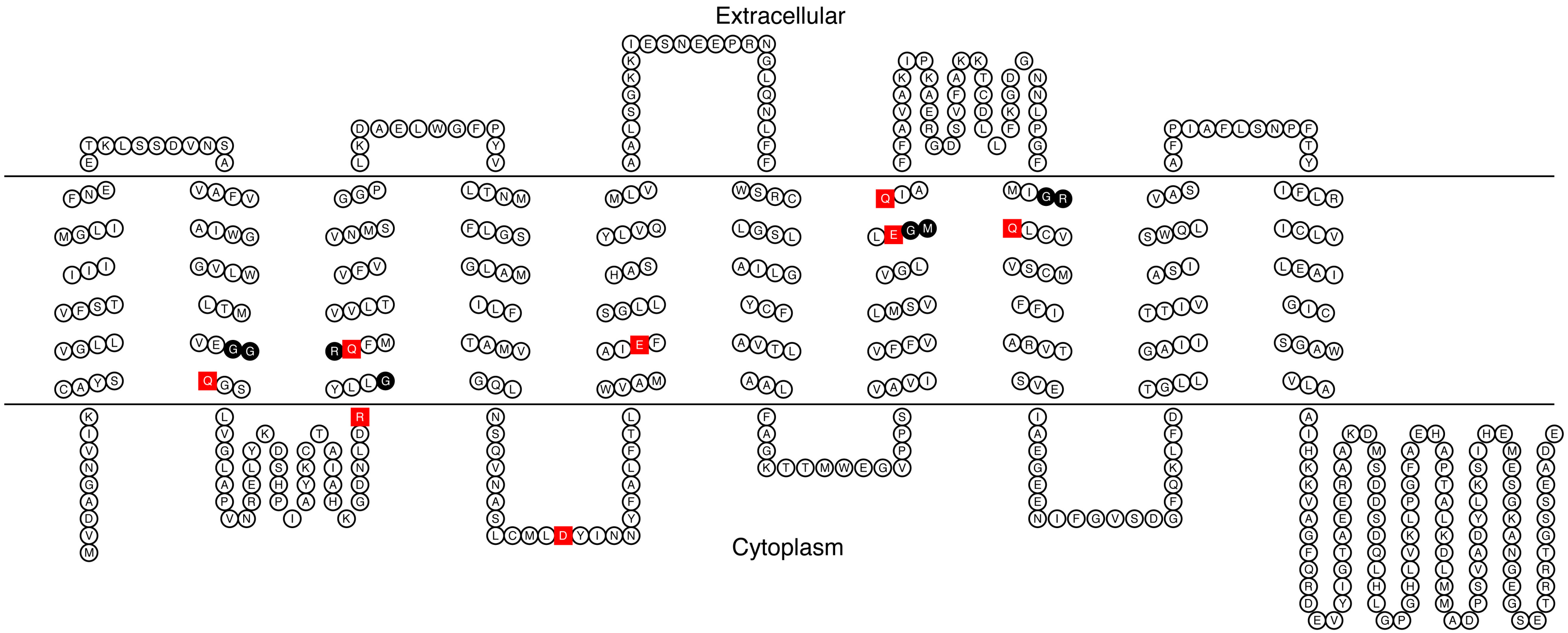

Supplementary Figure 3. Topology diagram of PtSIT1. The four conserved GXQ motifs are highlighted in black. Residues mutated in this study are highlighted in red.

4. SEDIMENTATION DISPERSITY ASSAY

The results of the sedimentation dispersity assay are shown in Supplementary Figure 4. NaSIT and PtSIT2 both had a broad solubility profile, being soluble in all detergents tested except BigCHAP, OG and OTG. In the case of NaSIT, greatest stability was in the detergents FC-12 and FC-14. PtSIT2 showed a more extended stability profile, although again the fos-cholines gave the highest yields of stable protein. PtSIT1 and CfSIT2 were essentially insoluble in all of the detergents tested except the fos-choline series, and were stable in the same.

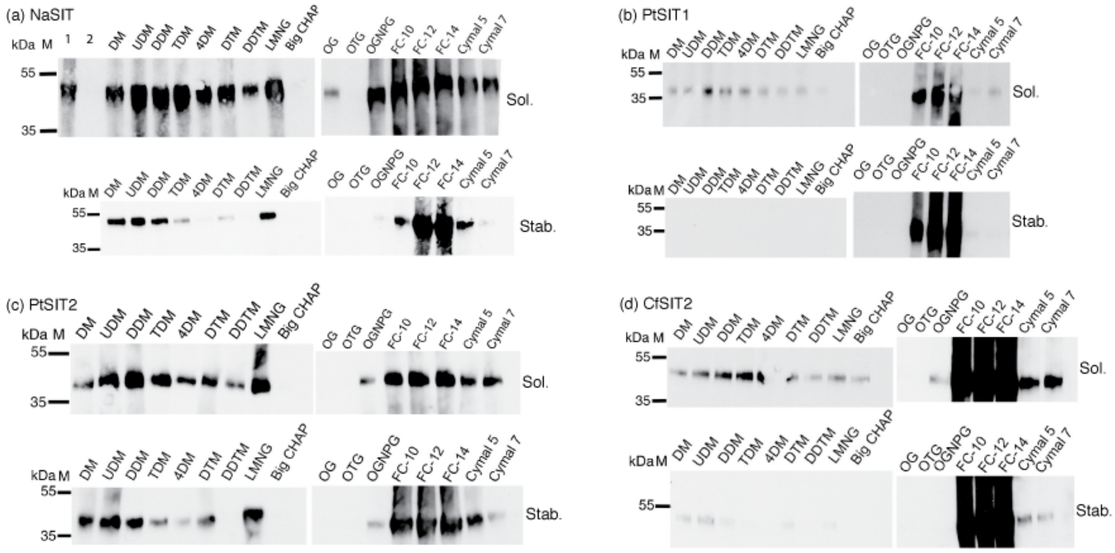

Supplementary Figure 4. Centrifugal sedimentation assays to determine SIT solubility (*Sol.*) and propensity to aggregation, which we term here stability (*Stab.*). (**a**) Assay results for NaSIT, with lanes 1 and 2 respectively being uncentrifuged and no detergent controls. These lanes are omitted from the results for (**b**) PtSIT1, (**c**) PtSIT2 and (**d**) CfSIT2. All images are qualitative Western blots with anti-V5-HRP, subject to different exposure times. Gel mobility is determined relative to molecular weight markers (*M*). Blots are overdeveloped in (**d**) to allow visualization of weaker bands.

5. PURIFICATION OF NaSIT, PtSIT1, PtSIT2 and CfSIT2

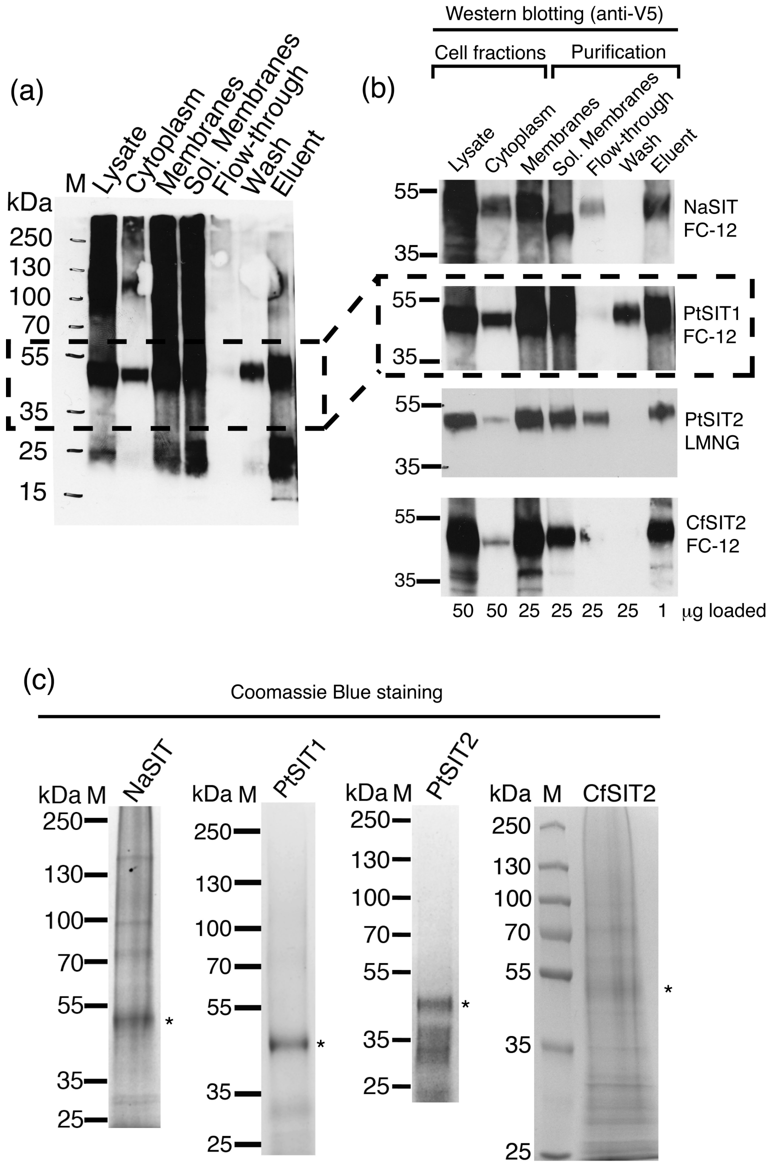

Supplementary Figure 5. Purification of selected SITs. (**a, b**) Western blotting was used to track the fate of the SITs during cell fractionation and affinity purification. The total protein loaded in each lane was the same for each gel as shown, although the independent blots are developed at different exposure times for presentation. Panel (**a**) shows the uncropped blot for PtSIT1. The band at ~25 kDa is full-length PtSIT1 (see Fig. 3 and accompanying text). (**c**) SDS-PAGE of purified SITs stained with Coomassie Brilliant Blue. Bands corresponding to purified SITs are marked (*). Data for NaSIT and PtSIT1 are also presented in Fig. 1 of the manuscript and are shown here to allow direct comparison. See also Supplementary Note 2.

6. CHARACTERIZATION OF PTSIT1 AND SIT HOMOLOGUES

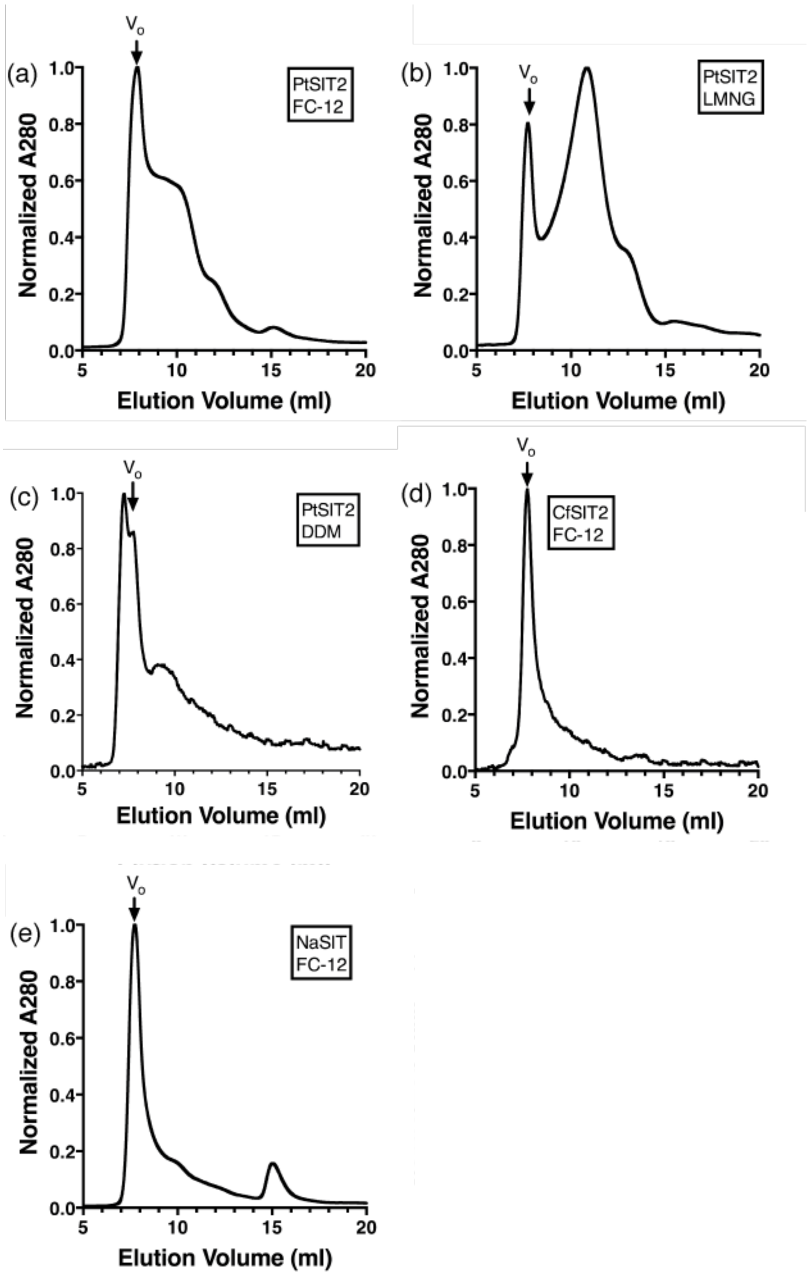

Supplementary Figure 6. Size exclusion chromatography of highly-expressing SIT homologues other than PtSIT1. Chromatograms show substantial heterogeneity, with broad peaks that are unlikely to resolve to single protein species and extensive aggregation to homooligomers of >650 kDa that elute in the void volume (*Vo*). (**a-c**) PtSIT2 in FC-12, LMNG and DDM. (**d**) CfSIT2 in FC-12. (**e**) NaSIT in FC-12.

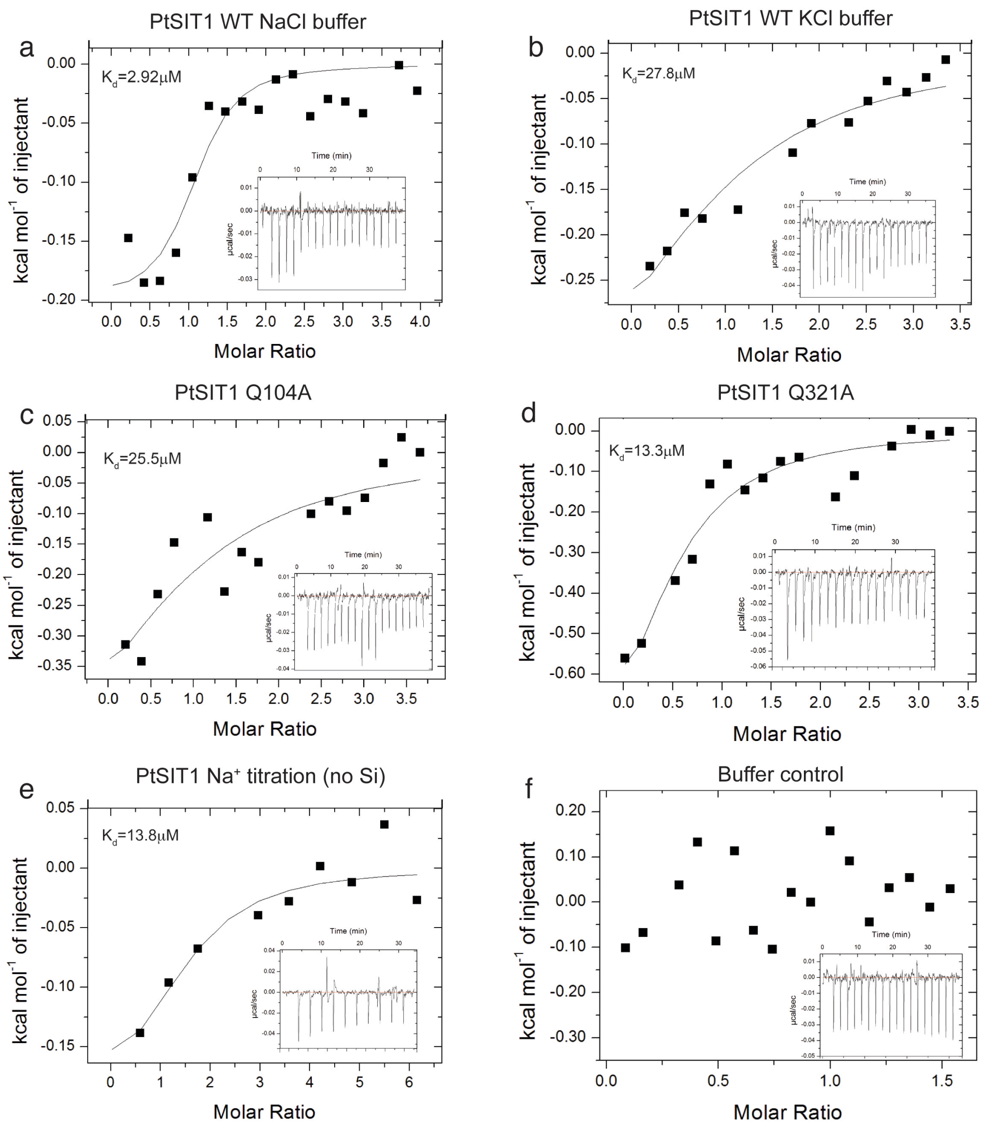

Supplementary Figure 7. Isothermal titration calorimetry. (**a-e**) Raw data for PtSIT1 in the presence and absence of buffer sodium and of PtSIT1 mutant proteins as shown. (**f**) Negative control showing injection of identical buffer without silicic acid into protein solution. Panel (**a**) is also presented as part of Fig. 2c and is shown here to allow direct comparison.

7. ZINC SILICATE FLUORESCENCE

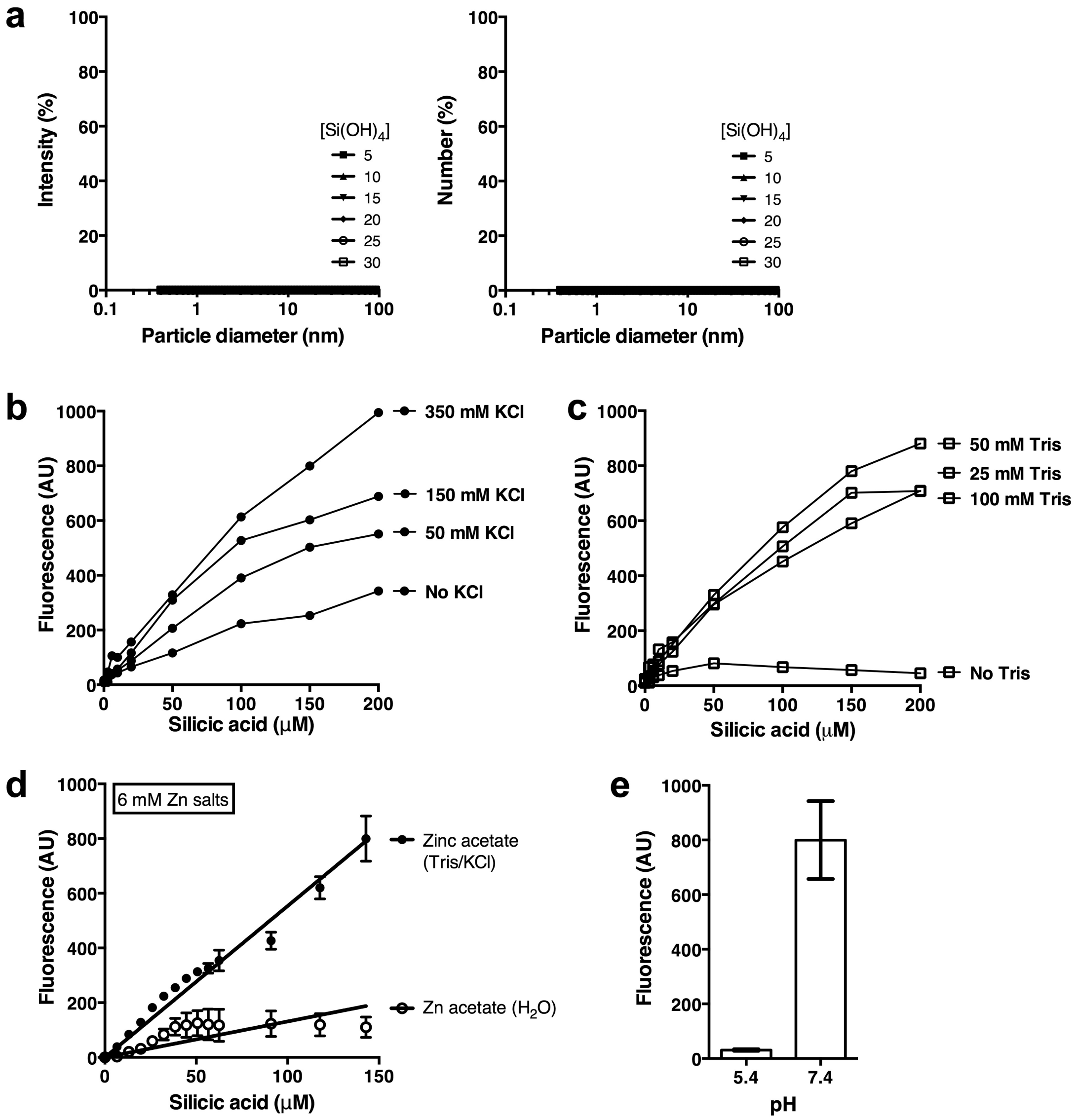

Supplementary Figure 8. Characterization of fluorescent zinc silicates under different conditions. (**a**) Zinc silicates at low silicic acid concentrations do not form particles that can be detected by dynamic light scattering. Data are shown either by raw intensity (*left*) or particle number (*right*), truncated at 100 nm for presentation to omit a buffer background peak present in all samples at ~300 nm. (**b-d**) Zinc silicate fluorescence is substantially enhanced by salts and Tris buffer. (**e**) Zinc silicate fluorescence is abolished at low pH.

8. RECONSTITUTION INTO PROTEOLIPOSOMES

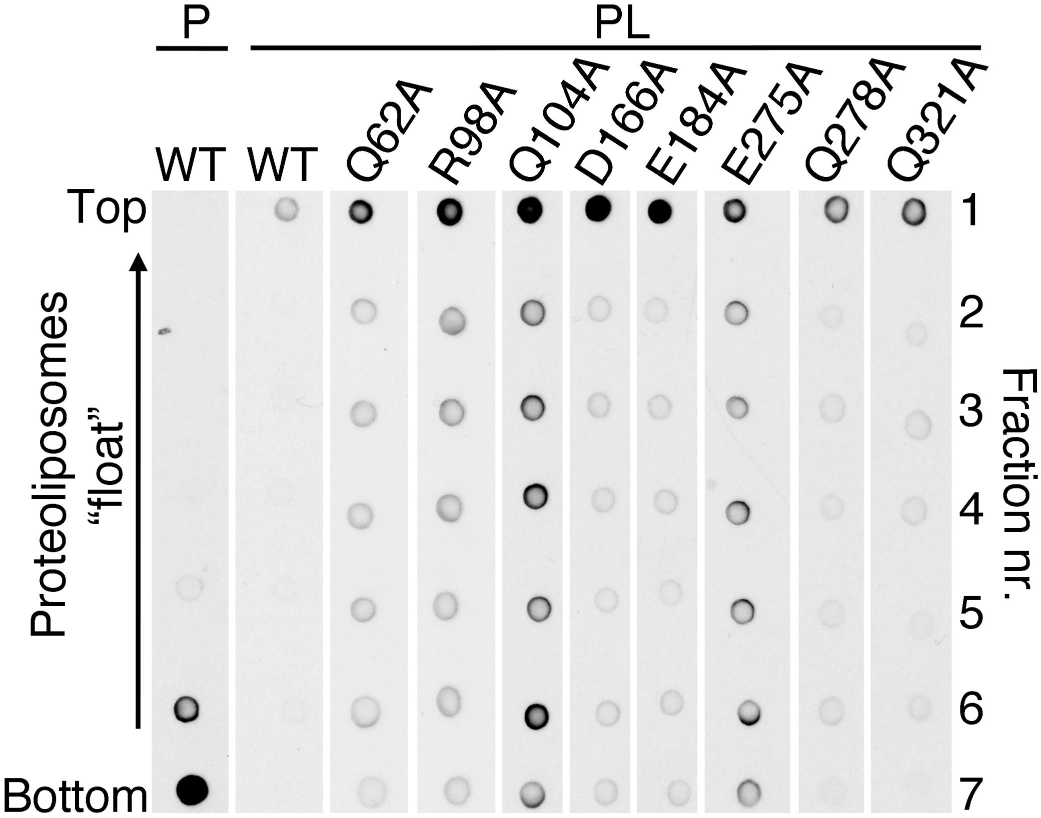

Supplementary Figure 9. Discontinuous sucrose density gradients confirm reconstitution of SITs into proteoliposomes. Qualitative dot blots show that protein-only controls (*P*) remain at the bottom of the gradient while proteoliposomes (*PL*) migrate to the top.

9. BIOPHYSICAL CHARACTERIZATION OF PtSIT1 MUTANTS

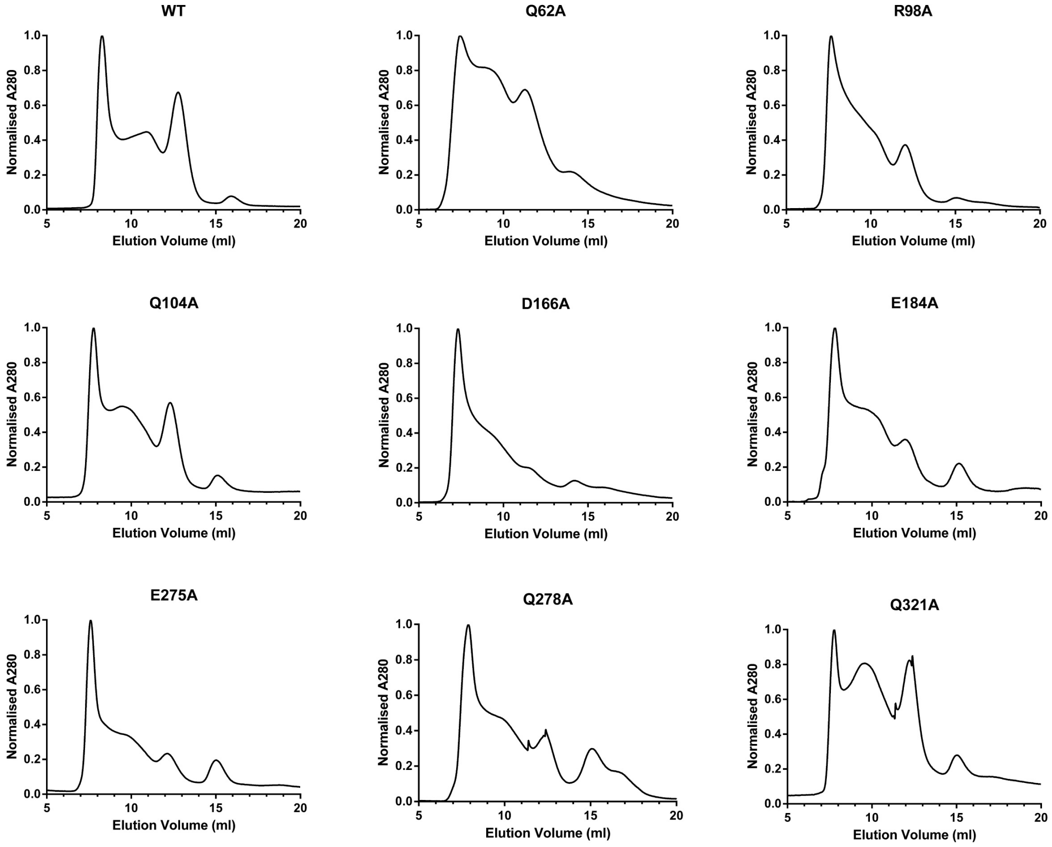

Supplementary Figure 10. Size-exclusion chromatography of PtSIT1 mutants in FC-12. R98A, Q104A and Q321A are reasonably similar to WT while other mutants show increased heterogeneity and non-specific aggregation.

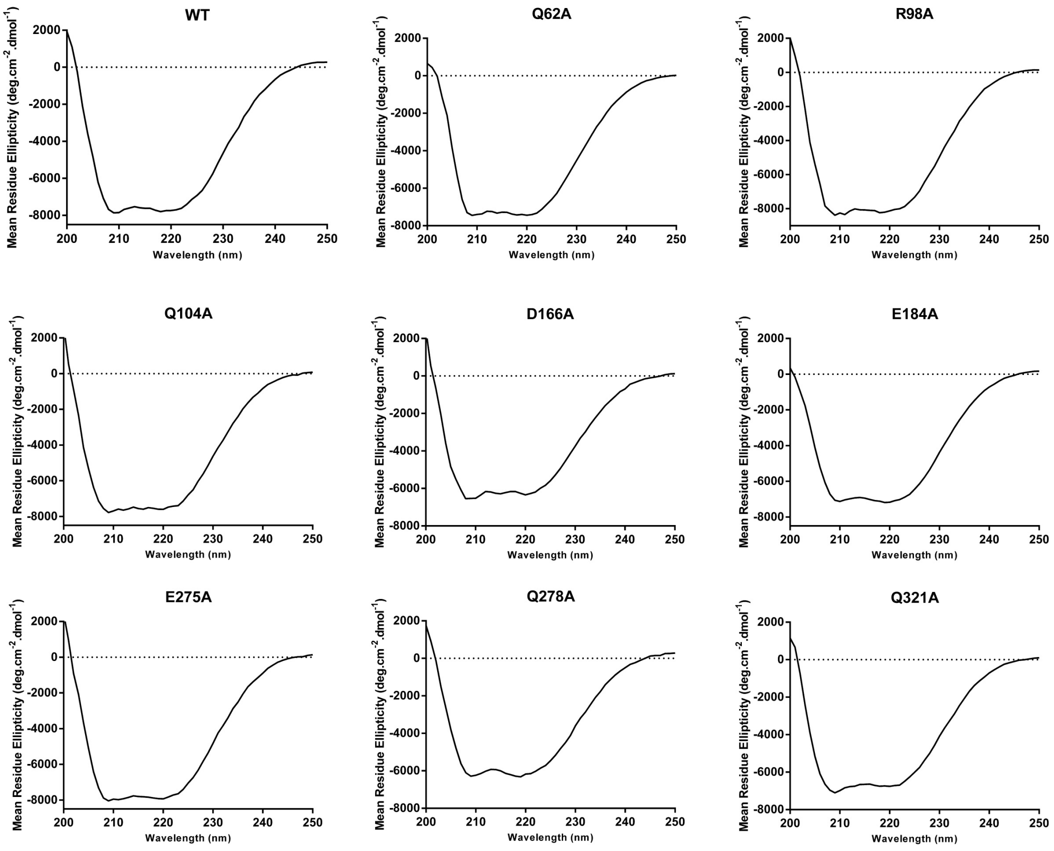

Supplementary Figure 11. Circular dichroism spectroscopy of PtSIT1 mutants in FC-12. All mutants had similar secondary structure content to WT except D166A, E184A and Q278A, in which ellipticity was slightly reduced.

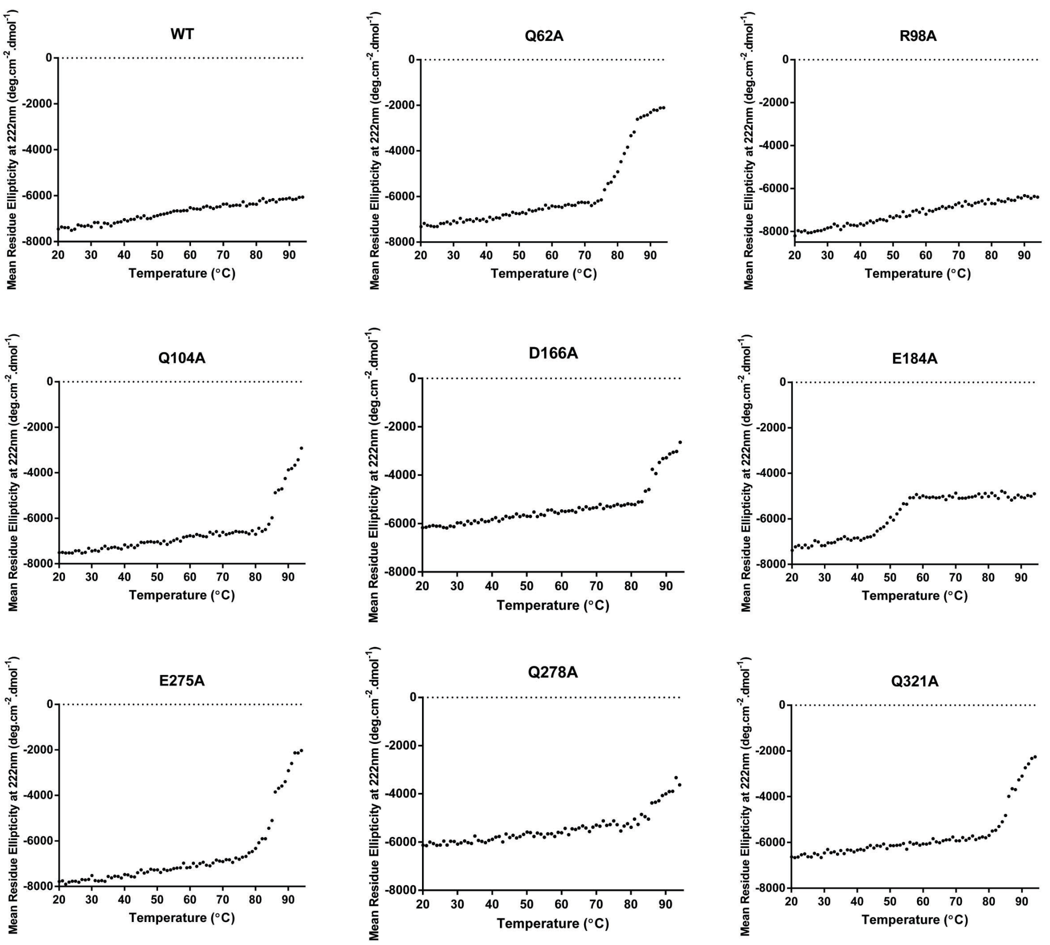

Supplementary Figure 12. Thermal melts of PtSIT1 mutants in FC-12. R98A was similar to WT in not showing a cooperative transition over the temperatures surveyed. E184A was apparently destabilized significantly, with T_m ~50°C. All other mutants showed a major cooperative transition with T_m ~85°C.

Supplementary Table 1. Results of isothermal titration calorimetry with PtSIT1.

**Protein (buffer salt)**	**Titrant**	**K_d (μM)**	**N**	**ΔH (kcal mol^{-1})**	**TΔS* (kcal mol^{-1})**	**ΔG (kcal mol^{-1})**
WT (NaCl)	$Si(OH)_4$	2.9 ± 2.3†	1.1 ± 0.1	-0.2 ± 0.03	7.2	-7.4 ± 1.1
WT (KCl)	$Si(OH)_4$	27.8 ± 6.9	1.0‡	-0.5 ± 0.1	5.6	-6.1 ± 0.7
WT (KCl)	NaCl	13.8 ± 16.1	1.4 ± 0.7	-0.2 ± 0.1	6.3	-6.5 ± 3.3
Q104A (NaCl)	$Si(OH)_4$	25.5 ± 11.0	1.0‡	-0.7 ± 0.2	5.4	-6.1 ± 1.7
Q321A (NaCl)	$Si(OH)_4$	13.3 ± 8.0	0.5 ± 0.2	-1.1 ± 0.6	5.4	-6.5 ± 3.5

*T = 293K

†Errors represent ± s.e.m from curve fitting to a single-site equation

‡Stoichiometry constrained to 1.0 to permit curve fitting

Supplementary Note 1. Multiple sequence alignment was carried out with ClustalOmega (http://www.ebi.ac.uk/Tools/msa/clustalo/)[1]. As expected from previous observations[2,3] the SITs showed somewhat higher sequence conservation between predicted transmembrane regions, including the conserved GXQ motifs, and greater divergence within the loops and termini. The protein sequences were between 506-599 amino acids in length and within our small dataset they cluster into three groups. The first 'short' group, comprising TpSIT1, PtSIT1, PtSIT2, ToSIT and ScSIT, all had sequences of 514 ± 8 (mean ± s.d.) amino acids. The second 'intermediate' grouping, comprising TpSIT3, CfSIT1-3 and NaSIT, had mean average sequence lengths of 552 ± 9 amino acids. PtSIT3 appears to sit outside these two simple groupings, being longer at 599 amino acids. The variations in sequence length arose from additional amino acids at either the N-terminal (TpSIT3 and PtSIT3) or the C-terminal (CfSIT1-3 and NaSIT). These C-terminal regions, which are rich in A, K, L and E, were previously identified as having the potential for forming intracellular coiled-coils that could be important in protein-protein interactions[2]. The N-terminus of PtSIT3 appeared to be particularly enriched for A, D, E, P, S and T with these six amino acids accounting for 79 of the first 140 residues (56%). There was no obvious similarity between the N-terminal extensions of PtSIT3 and TpSIT3.

Supplementary Note 2. We purified detergent-solubilized NaSIT, PtSIT1, PtSIT2 and CfSIT2 by using Ni^{2+} affinity chromatography against the C-terminal His_{10}-tag. Western blotting was used to determine the cellular localization of each of the recombinant SITs and all were found to be strongly associated with the sedimenting membrane fraction (Supplementary Figure 5a). Western blotting was also used to follow protein purification. After solubilization, all of the His-tagged proteins bound tightly to a Ni^{2+} resin (depleted from the column flow-through in Supplementary Figure 5). Proteins remained bound during stringent washing and were ultimately eluted from the column by competition with imidazole. Each of the purified recombinant SITs was visualized by staining SDS-PAGE gels with Coomassie Brilliant Blue (Supplementary Figure 5b). NaSIT and PtSIT1 both gave clear bands at the expected apparent molecular weight, although NaSIT appeared to have additional contaminant proteins at higher

molecular weights. PtSIT2 showed substantial contamination from other bands at lower molecular weights that do not appear on Western blots. These additional bands could represent proteolysis products where the C-terminal epitope is digested. CfSIT2 ran as a smear but did show an indistinct band at the expected apparent molecular weight. Total yields of CfSIT2 and NaSIT were ~0.2 mg protein litre^{-1} yeast culture, consistently lower than PtSIT1 and PtSIT2, which were typically 0.5 mg protein litre^{-1} yeast culture.

Supplementary References

1 Sievers, F. *et al.* Fast, scalable generation of high-quality protein multiple sequence alignments using Clustal Omega. *Mol Systems Biol* **7**, 539 (2011).
2 Thamatrakoln, K., Alverson, A. J. & Hildebrand, M. Comparative sequence analysis of diatom silicon transporters: towards a mechanistic model of silicon transport. *J. Phycol.* **42**, 822-834 (2006).
3 Marron, A. O. *et al.* A family of diatom-like silicon transporters in the siliceous loricate choanoflagellates. *Proc R Soc B* **280**, 20122543 (2013).
